# Encapsulation of *Olea europaea* Leaf Polyphenols in Liposomes: A Study on Their Antimicrobial Activity to Turn a Byproduct into a Tool to Treat Bacterial Infection

**DOI:** 10.1021/acsami.4c13302

**Published:** 2024-12-04

**Authors:** Giuliana Prevete, Enrica Donati, Anna Paola Ruggiero, Silvia Fardellotti, Laura Lilla, Valentina Ramundi, Isabella Nicoletti, Francesca Mariani, Marco Mazzonna

**Affiliations:** † Institute for Biological Systems (ISB), Consiglio Nazionale delle Ricerche (CNR), Territorial Research Area Rome 1, Strada Provinciale 35d, no. 9, 00010 Montelibretti, Rome, Italy

**Keywords:** *Olea europaea* leaf extracts, polyphenols, liposomes, *Staphylococcus aureus*, antimicrobial acitivity, synergic antibacterial effect

## Abstract

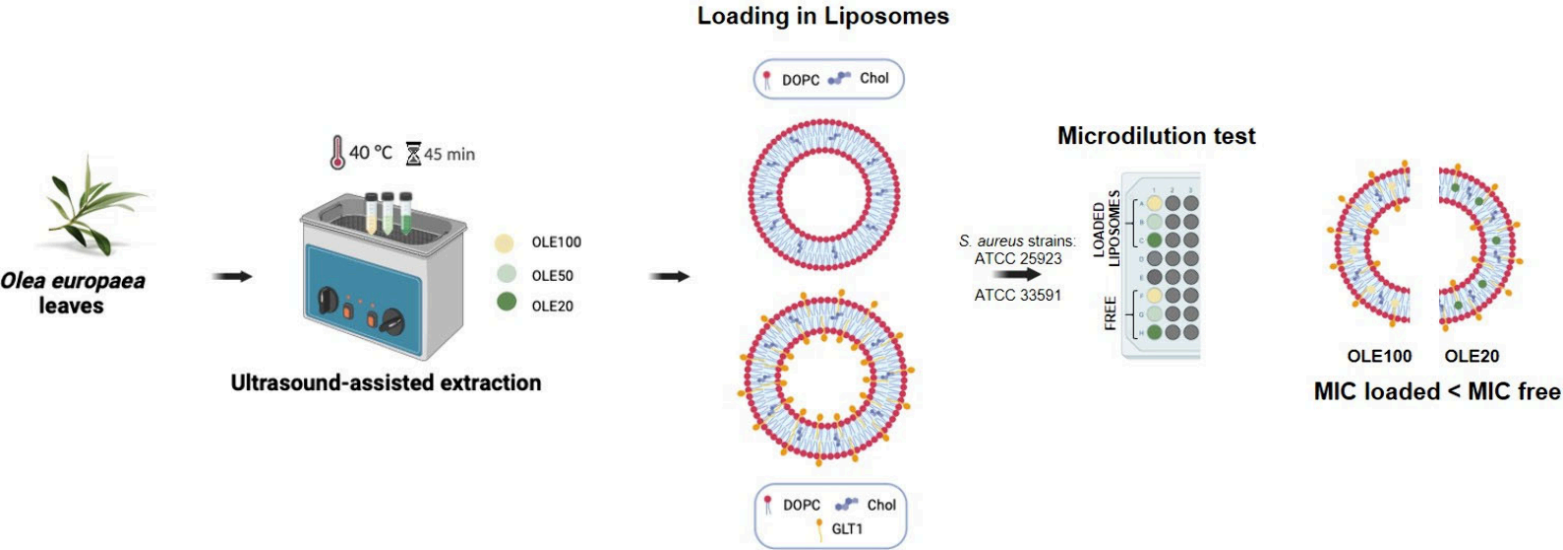

According to the innovative and sustainable perspective of the circular economy model, *Olea europaea* leaves, a solid byproduct generated every year in large amounts by the olive oil production chain, are considered a valuable source of bioactive compounds, such as polyphenols, with many potential applications. In particular, the following study aimed to valorize olive leaves in order to obtain products with potential antibacterial activity. In this study, olive leaf extracts, rich in polyphenols, were prepared by ultrasound-assisted extraction using green solvents, such as ethanol and water. The extracts were found to be rich in polyphenols up to 26.7 mg_GAE_/g_leaves_; in particular, hydroxytyrosol-hexose isomers (up to 6.6 mg/g_dry extract_) and oleuropein (up to 324.1 mg/g_dry extract_) turned out to be the most abundant polyphenolic compounds in all of the extracts. The extracts were embedded in liposomes formulated with natural phosphocholine and cholesterol, in the presence or in the absence of a synthetic galactosylated amphiphile. All liposomes, prepared according to the thin-layer evaporation method coupled with an extrusion protocol, showed a narrow size distribution with a particle diameter between 79 and 120 nm and a good polydispersity index (0.10–0.20). Furthermore, all developed liposomes exhibited a great storage stability up to 90 days at 4 °C and at different pH values, with no significant changes in their size and polydispersity index. The effect of the encapsulation in liposomes of *O. europaea* leaf extracts on their antimicrobial activity was examined *in vitro* against two strains of *Staphylococcus aureus*: ATCC 25923 (wild-type strain) and ATCC 33591 (methicillin-resistant *S. aureus*, MRSA). The extracts demonstrated good antimicrobial activity against both bacterial strains under investigation, with the minimum inhibitory concentration ranging from 140 to 240 μg_extract_/mL and the minimum bactericidal concentration ranging from 180 to 310 μg_extract_/mL, depending on the specific extract and the bacterium tested. Moreover, a possible synergistic effect between the bioactive compounds inside the extracts tested was highlighted. Notably, their inclusion in galactosylated liposomes highlighted comparable or slightly increased antimicrobial activity compared to the free extracts against both bacterial strains tested.

## 1 Introduction

The *Olea europaea* tree, belonging to the *Oleaceae* family and *Olea* genus, is one of the most emblematic fruit trees of the whole Mediterranean area. Since antiquity, olive trees have been cultivated to produce olive oil and compounds suitable for beneficial and medicinal purposes,^1^ due to the presence of phenolic bioactive compounds identified in many tree components and byproducts.

In particular, *O. europaea* leaves, produced in large amount during the harvesting of olive fruit and the pruning of olive trees, are considered a valuable source of polyphenols, which represent one of the most important secondary metabolite categories produced by plants as a defense mechanism against pathogens, parasites, herbivores, and many stress triggers. Moreover, the amounts of polyphenols produced are strictly related to the type of cultivar, the state of soil hydration, and the condition of plant growth such as temperature, soil properties, light, and irrigation.^2,3^

Olive leaf extracts (OLEs) contain many of these bioactive polyphenols, exhibiting several health benefits such as antioxidant, antiinflammatory, antitumor, hepatoprotective, neuroprotective, immune-stimulant, antiaging, antiviral, and antimicrobial properties.^4,5^

Regarding the antimicrobial activity, OLEs have been proven to be active against many bacteria species, both Gram-positive and Gram-negative, such as *Escherichia coli*,^6,7^*Pseudomonas aeruginosa*,^6,8^*Staphylococcus aureus*,^6,7,9−12^*Bacillus subtilis*,^6^ and *Klebsiella pneumoniae*,^6^ thanks to their ability to affect a multitude of bacterial molecular target.^13^

Thereby, the recovery and reuse of this byproduct, which represents an economic and environmental problem for olive growers, can be an example of a circular economy, which aims to turn biomass waste and residues into valuable products, in order to minimize waste production. In this perspective, olive leaf extraction is the key step in the recovery of bioactive compounds, which can be achieved according to traditional techniques^14^ and innovative green methods.^15−17^

Nowadays, commercial applications of OLEs are mostly limited to folk medicine,^18,19^ and although many efforts have been made to extend their use from traditional to pharmaceutical applications, their utilization in modern medicine is limited by several challenges, such as the complex composition in active molecules of their extracts, as well as the stability and bioavailability of these molecules.

Generally, the study of the therapeutic and pharmacological properties of plant extracts is limited to determination of the main bioactive compounds with the aim of identifying a candidate that can be used for drug development. Nevertheless, botanical extracts activity is very often due to the combined action of different molecules present in them, which can be synergic or antagonistic.^20−22^

Despite their health-promoting effects, the use of polyphenols for human health is limited by many physicochemical factors, affecting their specific low absorption rate and, consequently, their low bioavailability at the target site. This latter feature is mainly related to the low polyphenols solubility in aqueous media and biological fluids,^23^ poor stability in the gastrointestinal tract,^24^ low permeation on the surface of small-intestine epithelial cells, susceptibility to environmental factors (pH, enzymes, and oxygen), and extensive metabolic reactions.^25^

However, the bioavailability of polyphenols in humans can be improved by encapsulating them in appropriate delivery systems.^26−28^

Different methods for the encapsulation of OLEs have been reported in the literature, such as microencapsulation by freeze-drying,^29^ formation of inclusion complexes with cyclodextrin,^30^ encapsulation with sodium alginate by spray-drying,^31^ inclusion in alginate–chitosan copolymer microbeads by electrostatic extrusion,^32^ nanoencapsulation in W/O/W emulsions,^33^ and loading in liposomes.^9,34^

To ensure an appropriate encapsulation strategy, several factors must be considered such as the achievement of good encapsulation efficiency, the release profile of the encapsulated polyphenols, and the final particle size of the carrier system, which is usually around or below 100 nm for pharmaceutical purposes.^35^

Among all of the studied nanoparticle delivery systems, liposomes are considered the most promising and versatile for potential medical applications. In fact, compared to traditional drug-delivery systems, liposomes offer several advantages, including site-targeting, sustained or controlled release, protection of drugs from degradation and clearance, superior therapeutic effects, reduced toxic side effects, and versatility in encapsulating lipophilic, hydrophilic, and amphiphilic compounds. Additionally, their dimensions can be controlled, and they can be functionalized for targeted delivery.^36,37^

Evidence of liposomes enhancing the bioactivity and bioavailability of polyphenols has been reported by a number of researchers;^38^ moreover, the biological activity of polyphenols embedded in liposomes can be potentially enhanced or reduced by the encapsulation, as was already reported in the literature.^9,39−41^

From a circular economy perspective, the aim of this work is the recovery and valorization of olive leaves to obtain products with antibacterial activity. In fact, the combined action of the many different biomolecules contained in these extracts, exerted through different cellular mechanisms of action, could prevent the development of bacterial resistance to antibiotics, thus providing an alternative or complementary tool to treat infections with drug-resistant bacterial pathogens.

**Chart 1:**
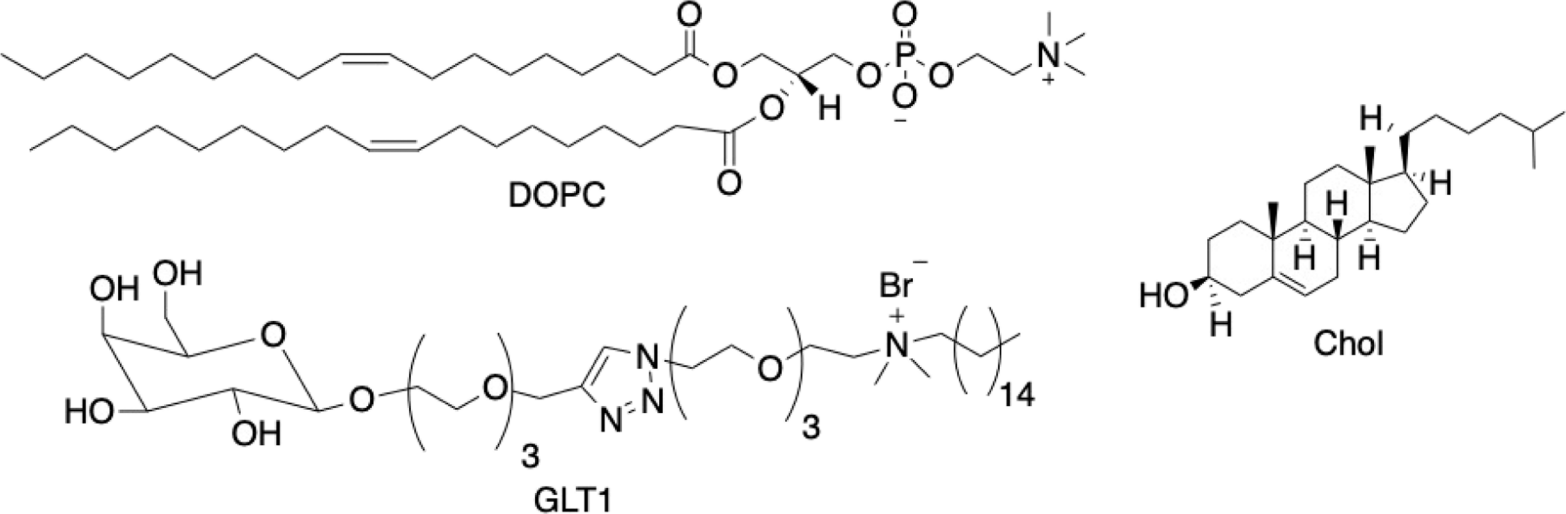
Lipid Components of Liposomes Developed

OLEs were prepared by ultrasound-assisted extraction (UAE) using different mixtures of green solvents, such as water and ethanol. The extracts produced were characterized in terms of yield of extraction, total phenolic content, and antioxidant capacity; moreover, the main phenolic compounds present in the extracts were identified and quantified by ultraperformance liquid chromatography (UPLC)–photodiode array (PDA)–mass spectrometry (MS) analysis. Afterward, dry extracts, or oleuropein (the main polyphenol present in OLEs), were loaded in liposomes formulated with a natural phospholipid, namely, 1,2-dioleoyl-*sn*-glycero-3-phosphocholine (DOPC) and cholesterol (Chol), in the presence or absence of a cationic galactosylated amphiphile (GLT1; Chart 1).

Liposomes were characterized in terms of the dimensions, polydispersity index (PDI), ζ potential, and entrapment efficiency (EE). Moreover, the liposome stability over time and at different pH values was evaluated, and the forced release of entrapped polyphenols over time was investigated.

Finally, the antimicrobial activities of both the extracts and the main polyphenols identified, free or loaded in liposomes, were investigated *in vitro* against two strains of *S. aureus*: ATCC 25923 (wild-type strain) and ATCC 33591 (methicillin-resistant *S. aureus*, MRSA).

## 2 Materials and Methods

### 2.1 Plant Material and Chemicals

Olive leaves from *O. europaea*, cultivar “Frantoio”, were picked up in Montelibretti (Rome, Italy) during the olive harvest period. The sampling concerned olive trees not subjected to any pest treatments, thereby avoiding any form of contamination. Immediately after sampling, the olive leaves were washed, crushed in a mortar under liquid nitrogen, and freeze-dried until a stable weight was obtained. Finally, the ground olive leaves were stored at −80 °C until further experiments.

1,2-Dioleoyl-*sn*-glycero-3-phosphocholine (DOPC) was purchased from Avanti Polar Lipids (Alabaster, AL). Cholesterol (Chol; purity 99%), hydroxytyrosol (4-dihydroxyphenylethanol, purity ≥98%), oleuropein [(2*S*,3*E*,4*S*)-3-ethylidene-2-(β-d-glucopyranosyloxy)-3,4-dihydro-5-(methoxycarbonyl)-2*H*-pyran-4-acetic acid 2-(3,4-dihydroxyphenyl) ethyl ester, purity ≥80%], verbascoside (purity ≥99%), trolox [(±)-6-hydroxy-2,5,7,8-tetramethylchromane-2-carboxylic acid, purity ≥97%], 2,2′-azinobis(3-ethylbenzothiazoline-6-sulfonic acid diammonium salt (ABTS; purity ≥98%), Folin and Ciocalteu’s phenol reagent, potassium persulfate (purity >99%), sodium hydroxide (NaOH; purity 98%), phosphate-buffered saline (PBS; 0.01 M phosphate buffer, 0.0027 M KCl, and 0.137 M NaCl, pH 7.4, at 25 °C, prepared by dissolving 1 tablet in 200 mL of deionized water), cellulose dialysis membrane (D9527-100FT, molecular weight cutoff = 14 kDa), and chloroform (CHCl_3_; analytical grade) were purchased from Sigma-Aldrich (St. Louis, MO). Gallic acid (purity ≥98%), 4-hydroxyphenylacetic acid (purity ≥98%), and sodium carbonate (purity ≥98%) were purchased from Fluka Chemie GmbH (Buchs, Switzerland).

Methanol (MeOH), ethanol (EtOH), acetonitrile (ACN), and water (H_2_O), all HPLC-grade, were purchased from VWR International s.r.l. (Milan, Italy). Formic acid and hydrochloric acid (HCl; 37%) were supplied by Carlo Erba (Milan, Italy).

Muller–Hinton (MH) broth and MH agar were purchased from Fisher Scientific (Milan, Italy).

The galactosylated amphiphile GLT1 was synthesized according to a procedure reported in the literature.^42^

### 2.2 Preparation of OLEs

Aqueous and hydroalcoholic extracts from olive leaves were obtained by UAE using a bath sonicator (Elmasonic S 30 H). A total of 500 mg of olive leaves was extracted with 10 mL of different mixtures of solvents such as H_2_O (100%), EtOH/H_2_O (50:50 v/v), and EtOH/H_2_O (80:20 v/v), obtaining three different extracts identified as OLE100, OLE50, and OLE20, respectively. The ultrasonic extraction was carried out at 40 °C for 45 min. The extracts were then centrifuged (UNIVERSAL 320R, Hettich) at 4000 rpm for 10 min at 20 °C to remove the insoluble fraction, and the obtained supernatants were analyzed by both spectrophotometric and chromatographic methods.

### 2.3 Freeze-Drying Process

OLE100, OLE50, and OLE20 were freeze-dried using a FreeZone 7740030 (LabConco Corp.). Before being freeze-dried, EtOH was removed from OLE50 and OLE20 under vacuum by a rotary evaporator. For each extract, the yield of extraction [*R* (%)] was calculated as follows:
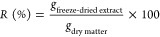
1where *g*_freeze-dried extract_ corresponds to the amount of dry extract obtained by lyophilization and *g*_dry matter_ corresponds to the amount of olive leaves used for the extraction.

### 2.4 Chemical Characterization of OLEs

#### 2.4.1 Total Phenolic Content (TPC)

The TPC of OLE100, OLE50, and OLE20 was evaluated by Folin–Ciocalteu assay.^43,44^ Briefly, 10 μL of OLE100, OLE50, or OLE20 was mixed with 50 μL of Folin–Ciocalteu reagent and 150 μL of 2% (w/v) Na_2_CO_3_, bringing the final volume of the solution to 1 mL with water. After 2 h of incubation in the dark at 25 °C, the absorbance was measured at 760 nm by a spectrophotometer (UV-2401PC, Shimadzu, Kyoto, Japan). The TPC of each extract was determined using gallic acid as the reference standard (calibration curve 0.025–2.0 mg/mL), and the results were expressed as milligrams of gallic acid equivalents per gram of extracted olive leaves (mg_GAE_/g_leaves_).

#### 2.4.2 Trolox Equivalent Antioxidant Capacity (TEAC)

The antioxidant capacity of OLE100, OLE50, and OLE20 was determined by TEAC assay following the reduction process of the ABTS radical cation (ABTS^•+^) to ABTS by reaction with antioxidant compounds.^45^

ABTS^•+^ was produced through the reaction between a 7 mM ABTS solution and 2.45 mM potassium persulfate in water, keeping the mixture under stirring overnight at room temperature in the dark before use. The stock ABTS^•+^ solution was diluted in EtOH to reach an absorbance of 0.70 ± 0.02 at 734 nm. Different volumes (2–10 μL) of the OLEs were added to 1 mL of the diluted ABTS^•+^ solution, and the reduction in absorbance was measured at 734 nm (UV-2401PC, Shimadzu, Kyoto, Japan) exactly 1 min after the initial mixing and up to 4 min.

The percentage of ABTS^•+^ inhibition (%_inhibition_) triggered by the antioxidant compounds present in OLEs was determined according to the following equation:
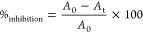
2where *A*_0_ is the absorbance recorded for the diluted ABTS^•+^ solution and *A*_t_ is the absorbance recorded after 1 or 4 min of reaction of the antioxidant compounds in OLEs with ABTS^•+^.

Trolox, a water-soluble analogue of vitamin E, was used as the reference standard, and the calibration curve (3.8–18.9 μM) was made by plotting %_inhibition_ as a function of the different concentrations of trolox added.

Finally, the %_inhibition_ determined for OLEs was expressed as millimoles of trolox equivalents per gram of extracted olive leaves (mmol_TE_/g_leaves_).

#### 2.4.3 Determination of the Main Phenolic Compounds in OLEs by UPLC–PDA–MS Analysis

Determination of the main phenolic compounds in the OLEs has been assessed by an UPLC Acquity H-Class Bio (Waters, Milford, MA) set up with a solvent mixing system, an autosampler, a thermostatically controlled column, and a PDA detector, directly coupled with an ion trap mass spectrometer (LXQ-MS System, Thermo Scientific, Waltham, MA). Phenolic compounds were separated using an Acquity UPLC HSS T3 column (1.8 μm, 150 × 2.1 mm i.d.; Waters, Milford, MA), maintaining the column temperature at 40 °C. A flow rate of 0.4 mL/min and an injection volume of 2 μL were used. The mobile phases were water [0.1% (v/v) formic acid, phase A] and ACN [0.1% (v/v) formic acid, phase B], changing the solvent gradient as follows: 0–3 min from 85% A and 15% B to 82% A and 18% B; 3–6.5 min from 82% A and 18% B to 77% A and 23% B; 6.5–10 min from 77% A and 23% B to 40% A and 60% B; 10–11 min from 40% A and 60% B to 100% B until the 22nd minute. The PDA detector recorded the spectra between 200 and 400 nm. The mass spectrometer operated in electrospray ionization (ESI) negative-ion mode using the following parameters: capillary temperature 275 °C; capillary voltage −10 V; spray voltage 3.60 kV; sheath gas flow 10 units; auxiliary gas flow 5 units. The instrument acquired data in the range *m*/*z* 100–700.

The UPLC method described above has been validated in terms of linearity, sensitivity, and precision. The limit of detection (LOD) and limit of quantification (LOQ) for each analyte were determined by gradual dilutions of the stock solutions by using signal-to-noise ratios of 3 and 10, respectively (Table S1).

Quantification of the main phenolic compounds in the OLEs was performed using the external calibration method. The calibration curves were obtained by analyzing standard solutions of different concentrations (*n* = 6) in triplicate in the following concentration ranges: hydroxytyrosol, 0.00011–1.1 mg/mL; verbascoside, 0.00036–0.84 mg/mL; oleuropein, 0.0038–1.9 mg/mL.

All of the calibration curves were linear in the concentration ranges studied, and the correlation coefficients (*R*^2^ factor) recorded were ≥0.9993 (Table S1).

Precision of the method was assessed in terms of repeatability by analyzing a solution containing hydroxytyrosol, verbascoside, and oleuropein. Intra- and interday precisions, expressed as the relative standard deviation (RSD), were evaluated by performing six consecutive injections of the same solution in the same day and over 3 days, respectively. In both cases, the RSD recorded was <2%.

### 2.5 Liposomes Preparation

Liposomes were formulated with a natural unsaturated phosphocoline (DOPC) and Chol in the presence or absence of the cationic galactosylated amphiphile GLT1.

Liposomes, both empty and loaded, were prepared according to the lipid film hydration protocol, coupled with the freeze–thaw procedure, and followed by an extrusion process.^46,47^

Briefly, a proper amount of lipid components was dissolved in CHCl_3_ (DOPC and Chol) and MeOH (GLT1) in a round-bottom flask and dried by rotary evaporation (Rotavapor R-200, BUCHI Labortechnik AG, Flawil, Switzerland) and then under high vacuum (5 h) to remove any traces of organic solvents and to obtain a thin lipid film.

Regarding the preparation of loaded liposomes, oleuropein (OLEUR) and OLEs were dissolved in MeOH and added to the lipid mixture, before film formation, to have a molar ratio of 1:8 OLEUR/lipids and a final ratio of 1:1 (w/w) lipids/dry extract, respectively.

Afterward, the film was hydrated with a PBS (150 mM) solution to give a liposomal suspension of 10 mM in total lipids concentration. The aqueous suspension was vortex-mixed to completely detach the lipid film from the flasks, and the obtained multilamellar vesicles (MLVs) were freeze–thawed five times, from liquid nitrogen to 50 °C. Size reduction of MLVs was carried out by extrusion (10 mL Liposome Extruder, Genizer, Irvine, CA) of liposomal dispersions, ten times under high pressure through a polycarbonate membrane with pore size of 100 nm (Whatman Nucleopore, Clifton, NJ) at a temperature higher than *T*_m_ to obtain small unilamellar vesicles. Finally, liposome purification from unentrapped polyphenols was performed by dialysis against PBS using a buffer volume equal to 25 times the total volume of the sample, under slow magnetic stirring.

### 2.6 Physicochemical Characterization of Liposomes

#### 2.6.1 Size and ζ-Potential Measurements

The size distribution, PDI, and ζ potential were determined using a Zetasizer Nano ZS (Malvern Instruments, Westborough, MA) equipped with a 5 mV He/Ne laser (λ = 632.8 nm) and a thermostated cell holder, setting the temperature at 25 °C for all of the measurements.

The particle size and PDI were measured through backscatter detection at an angle of 173°. The measured autocorrelation function was analyzed using the cumulant fit. The first cumulant was used to obtain the apparent diffusion coefficients, *D*, of the particles, further converted into apparent hydrodynamic diameters, *D*_h_, by using the Stokes–Einstein equation:
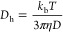
3where *k*_B_*T* is the thermal energy and η is the solvent viscosity.

To carry out the measurements, liposomal suspensions were diluted to 1 mM total lipid concentration in PBS (150 mM).

The ζ potential of liposome formulations was determined by electrophoretic light scattering (ELS) measurements, applying low voltages to avoid the risk of Joule heating effects. Analysis of the Doppler shift to determine the electrophoretic mobility was done by using phase-analysis light scattering (PALS),^48^ a method that is especially useful at high ionic strengths, where mobilities are usually low. The mobility μ of the liposomes was converted to ζ potential using the Smoluchowski relation ζ = μη/ε, where ε and η are the permittivity and viscosity of the solution, respectively.

To assess ELS measurements, liposomal suspensions were diluted to 1 mM in total lipids in diluted PBS (15 mM).

The data reported for *D*_h_, PDI, and ζ potential correspond to the average of three different independent experiments.

#### 2.6.2 Assessment of Liposomes Stability

The physical stability of OLEUR- and OLEs-loaded liposomes was evaluated over 90 days of storage at 4 °C protected from light sources, determining the vesicle size and PDI as previously described.

The stability of OLEUR- and OLEs-loaded liposomes was also investigated at different pH values, modified by adding appropriate volumes of HCl or NaOH aqueous solutions. The pH was set at the same values as those found in the digestive system.^49^ The average particle diameter and PDI were evaluated after incubation of liposomes at pH 5.7 for 1–3 min (mimicking mouth), at pH 2.9 for 30 min-3 h (mimicking stomach), at pH 6.4 for 3 h (mimicking intestine), and at pH 8 for 24 h (mimicking colon). All of the results collected were compared with those obtained at pH 7.4 in PBS (150 mM).^50^

#### 2.6.3 Determination of the EE

##### 2.6.3.1 Oleuropein-Loaded Liposomes

The content of OLEUR loaded in neutral and galactosylated liposomes was evaluated by UPLC–PDA analysis according to the procedure described below.

Before UPLC measurements, liposomes were properly diluted with MeOH to obtain their disruption and complete lipid solubilization. All samples were then filtered on poly(tetrafluoroethylene) (PTFE) membranes (4 mm × 0.2 μm; Sartorius) before injection.

According to the calibration curves reported in Table S1, the EE (%) of OLEUR loaded in liposomes was calculated using the following equation:
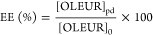
4where [OLEUR]_pd_ indicates the OLEUR concentration after the purification by dialysis and [OLEUR]_0_ corresponds to its concentration soon after extrusion.

##### 2.6.3.2 OLEs-Loaded Liposomes

The EE (%) of OLEs polyphenols embedded in neutral and galactosylated liposomes was determined by Folin–Ciocalteu assay (see above) by comparing the amount of polyphenolic compounds entrapped within the lipid vesicles with the amount measured in the dried extracts. Liposomal suspensions were diluted in MeOH (1:1 v/v) to break the lipid aggregates and enhance the release of embedded phenolic compounds. Moreover, the assay was carried out on empty neutral and galactosylated liposomes diluted with MeOH (1:1 v/v) to determine the contribution to Folin–Ciocalteu assay due to the lipid components. The results were expressed as micrograms of gallic acid equivalents (μg_GAE_), and the EE (%) was calculated using the following equation:
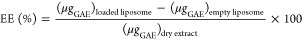
5where (μg_GAE_)_loaded liposome_, (μg_GAE_)_empty liposome_, and (μg_GAE_)_dry extract_ correspond to the micrograms of gallic acid equivalents obtained for extract loaded liposomes, empty liposomes, and unentrapped dry extract, respectively.

##### 2.6.3.3 Hydroxytyrosol-hexose Isomers, Verbascoside, and Oleuropein Encapsulated in OLEs-Loaded Liposomes

The amount of the main polyphenols identified in the OLEs, such as hydroxytyrosol-hexose (HOTyr-hexose) *isomer a* and *isomer b*, verbascoside (VERB), and OLEUR, was determined after the encapsulation of OLEs in both neutral and galactosylated liposomes, according to the UPLC-PDA analysis procedure reported above.

The EE (%) of the polyphenols entrapped in liposomes was calculated using the following equation:
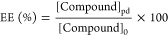
6where [Compound]_pd_ indicates the concentration of HOTyr-hexose *isomer a* and *isomer b*, VERB, or OLEUR from OLEs entrapped in liposomes determined after the dialysis purification and [Compound]_0_ corresponds to their concentration determined soon after the extrusion process.

#### 2.6.4 *In Vitro* Release Studies

##### 2.6.4.1 Oleuropein-Loaded Liposomes

The release of OLEUR from DOPC/Chol and DOPC/Chol/GLT1 liposomes was evaluated by a dialysis method (PBS volume 50 times the total volume of the sample), keeping the systems under stirring. Samples were collected every 1 h over a period of 24 h and analyzed by UPLC to study the releasing profile of OLEUR from liposomes. All of the collected liposomal aliquots were analyzed by UPLC after dilution with MeOH (1:1 v/v) and filtration by PTFE membranes (4 mm × 0.2 μm; Sartorius). The OLEUR content (mM) still embedded in liposomes at a specific time over a period of 24 h was determined by chromatographic analyses carried out as previously described.

##### 2.6.4.2 OLEs-Loaded Liposomes

The release of phenolic compounds from OLEs-loaded liposomes was determined by a dialysis method (PBS volume 50 times the total volume of liposome samples). Samples were collected every 1 h over a period of 24 h and analyzed by Folin–Ciocalteu assay (gallic acid used as the reference standard, calibration curve 10–2000 μg/mL) to investigate the releasing profile of the polyphenols embedded. All of the collected liposomal aliquots were analyzed after dilution with MeOH (1:1 v/v); afterward, the assay was assessed as described above. The phenolic content still encapsulated in liposomes determined at a specific time was expressed as micrograms of gallic acid equivalents per milliliter (μg_GAE_/mL).

### 2.7 *In Vitro* Antimicrobial Activity: Determination of the Minimum Inhibitory Concentration (MIC) and Minimum Bactericidal Concentration (MBC)

The microdilution method^51^ was used to investigate the *in vitro* antimicrobial activity of HOTyr, VERB, OLEUR, and OLEs, free or loaded in liposomes, against two strains of *S. aureus*: ATCC 25923 (wild-type strain) and ATCC 33591 (MRSA).

Gentamicin was tested as a control against both bacterial strains at a concentration of 5 μg/mL, showing a bactericidal effect against the ATCC 25923 strain and an inhibitory effect on the ATCC 33591 strain, as was already reported in the literature.

Besides, the activity of empty liposomes was also evaluated against both bacterial strains.

An overnight culture of each bacterial strain was prepared in MH broth and incubated at 37 °C. Afterward, the bacterial inoculum was diluted with MH broth, by measurement of 10-fold serial dilutions at 600 nm (Shimadzu UV-2401PC), to give a bacterial suspension containing approximately (2–8) × 10^5^ CFU/mL.

The diluted culture was aliquoted in a 96 well/plate flat bottom, and the antimicrobial agent, free or embedded in neutral or galactosylated liposomes, was added, in triplicate, at different concentrations. Then, the 96 wells/plates were incubated overnight at 37 °C. At the end of the incubation period, the plates were checked, and all of the transparent wells, likely corresponding to the MIC values, were streaked on a fresh MH agar plate and kept at 37 °C for 24 h. Growth inhibition in each 96 well/plate was compared to the growth positive control of each bacterial strain tested. Finally, MH agar plates were observed, and those showing bacterial growth were annotated as MIC while those showing nonbacterial growth were annotated as MBC.

## 3 Results and Discussion

### 3.1 Preparation of OLEs

OLEs were obtained by UAE, a versatile green technique for extracting bioactive compounds from plants, applicable at both laboratory and industrial scales. Compared to traditional solid–liquid extraction methods, UAE typically enables the successful extraction of natural products within minutes due to various ultrasound effects that lead to cell wall disruption, enhancing the mass transfer and release of bioactive compounds. Additionally, UAE allows for the use of less toxic solvents and a reduction in their consumption.^52^

Bioactive compounds from olive leaves were extracted using mixtures of H_2_O and EtOH in different ratios. These green solvents, in addition to be biocompatible, are able to produce extracts rich in polyphenols.^53−55^

UAE was carried out for 45 min at 40 °C, following preliminary studies that identified this extraction time as the optimal one (Table S2).

Three different extracts were produced using 100% water, 50:50 (v/v) EtOH/H_2_O, and 80:20 (v/v) EtOH/H_2_O as extracting solvents, and they were named OLE100, OLE50, and OLE20, respectively. OLEs were characterized in terms of the yield of extraction, TPC, and antioxidant activity (TEAC; Table 1).

**Table 1:** Yield of Extraction, TPC, and Antioxidant Activity (TEAC) of OLEs

extract	yield of extraction (%)	TPC (mg_GAE_/g_leaves_)	TEAC *t*_1 min_ (mmol_TE_/g_leaves_)	TEAC *t*_4 min_ (mmol_TE_/g_leaves_)
OLE100	33 ± 1	17.9 ± 0.6	0.16 ± 0.02	0.17 ± 0.03
OLE50	40 ± 2	24 ± 3	0.28 ± 0.02	0.31 ± 0.03
OLE20	41 ± 1	26.7 ± 0.8	0.34 ± 0.03	0.39 ± 0.05

### 3.2 Yield of Extraction

In order to determine the extraction yield, OLEs were freeze-dried also because the corresponding dry extracts are more stable, less degradable, and easier to handle compared to the liquid extracts.

For each extract, the extraction yield (%) was calculated as the percentage ratio between the weight of the freeze-dried extract and the weight of the olive leaves used in the extraction process. According to the data reported in Table 1, the extraction yields obtained for OLE50 and OLE20 are comparable to each other and higher than those achieved for OLE100.

### 3.3 TPC by Folin–Ciocalteu Assay

The TPC of OLE100, OLE50, and OLE20 was evaluated by Folin–Ciocalteu assay, using gallic acid as the reference standard. This assay is widely used for determination of the total phenols in plants extracts because it is convenient, quite easy to perform, and reproducible.^56^

TPC is expressed as gallic acid equivalents, and the results are reported as milligrams of gallic acid per gram of olive leaves extracted (mg_GAE_/g_leaves_). According to the results shown in Table 1, the TPC increases by increasing the percentage of EtOH in the mixture of extracting solvents, ranging from 17.9 mg_GAE_/g_leaves_ for OLE100 to 26.7 mg_GAE_/g_leaves_ for OLE20, in accordance with the results reported in the literature.^57,58^

It is worth highlighting that, although the extraction yields for OLE50 and OLE20 are comparable, the TPC is higher for OLE20, suggesting that a greater extraction yield does not directly result in a higher TPC.

### 3.4 Antioxidant Capacity by TEAC Assay

The antioxidant capacity was evaluated by TEAC assay based on the reaction between ABTS^•+^ and polyphenols contained in the OLEs.

ABTS^•+^ is a stable radical cation and a blue-green chromophore with a maximum absorbance at 734 nm. Its absorption at 734 nm decreases in the presence of antioxidant compounds able to quench it through a direct reduction by electron transfer or by hydrogen-atom transfer. Therefore, this assay is widely used to evaluate the antioxidant properties of many compounds, food, or plant matrixes.^59^

The antioxidant capacity of OLEs was determined at two different reaction times (1 and 4 min), following an end-point procedure reported in the literature.^45^

The results, expressed as millimoles of trolox equivalents per gram of olive leaves extracted (mmol_TE_/g_leaves_) in Table 1, displayed that the extract prepared using the highest percentage of EtOH in the mixture of extracting solvents (OLE20) shows the highest antioxidant capacity, which in detail doubles upon going from OLE100 to OLE20, with a trend similar to that recorded for the TPC.

### 3.5 Identification and Quantification of Phenolic Compounds by UPLC–PDA–MS

In order to identify and quantify the main polyphenols present in the OLEs, an UPLC–PDA–MS method was developed by analyzing a mixture of several analytical standards representing the main phenolic compounds that can usually be found in *O. europaea* leaves (Figure S1).

Initially, to obtain preliminary information on the predominant *m*/*z* ratios observed during chromatographic elution, a full-scan MS acquisition (*m*/*z* 100–700, in negative mode) was performed in combination with UPLC–PDA analysis. Subsequently, by the generation of extracted ion chromatograms and by a comparison of their retention times (RTs) and UV and MS spectra with those of the reference standards, peaks with pseudomolecular ions *m*/*z* 623 (RT = 6.45 min) and *m*/*z* 539 (RT = 9.08 min) were identified as VERB and OLEUR, respectively.

Specifically, OLEUR was found to be the most abundant polyphenol present in the OLE50 and OLE20 extracts and one of the most abundant in OLE100, whereas very small amounts of VERB were found in all of the OLEs (Figure S2).

Furthermore, UPLC–PDA–ESI-MS analysis of the OLEs revealed the presence of hydroxytyrosol attached to a six-carbon-atom sugar in all of the extracts. In particular, two structural isomers of this compound characterized by a pseudomolecular ion [M – H]^−^ with *m*/*z* 315 but with different RTs (respectively 1.29 and 1.56 min; Figure S3) were detected. The presence of these types of compounds in olive leaves is already known in the literature.^60,61^ In the following sections, these two isomers are referred to as HOTyr-hexose “*isomer a*” (RT = 1.29 min) and “*isomer b*” (RT = 1.56 min).

The UV–vis and MS spectra recorded for HOTyr-hexose *isomer a* and *isomer b*, VERB, and OLEUR are reported in Figures S4–S11.

The amounts of HOTyr *isomer a* and *isomer b*, VERB, and OLEUR in the OLEs were determined by UPLC–PDA analysis by using the external calibration method. The calibration curves were obtained using the corresponding analytical standards for VERB and OLEUR, whereas because the corresponding reference standards were not available for the two glycosylated HOTyr isomers to quantify them, the analytical standard of HOTyr was used.

In Table 2, the collected results are expressed as milligrams of compound per milliliter of extract (mg/mL_extract_), milligrams of compound per gram of extracted olive leaves (mg/g_leaves_), and milligrams of compound per gram of dry extract (mg/g_dry extract_). It is worth noting that, among the compounds identified, OLEUR is the most abundant one in all of the extracts. In particular, its amount increases as a function of the percentage of EtOH present in the extraction solvent, from 24.1 mg/g_dry extract_ for OLE100 to 324.1 mg/g_dry extract_ for OLE20. Instead, VERB is the least abundant in both hydroalcoholic extracts, and it is not quantifiable (<LOQ) in OLE100. Furthermore, HOTyr-hexose *isomer a* and *isomer b* are present in all of the OLEs in comparable amounts.

**Table 2:** Amounts of HOTyr-hexose *Isomer a* and *Isomer b*, VERB, and OLEUR in the OLEs Produced

	compound	mg/mL_extract_	mg/g_leaves_	mg/g_dry extract_
OLE100	HOTyr-hexose *isomer a*	0.04 ± 0.01	0.7 ± 0.1	1.6 ± 0.1
	HOTyr-hexose *isomer b*	0.10 ± 0.01	2.1 ± 0.2	5.0 ± 0.4
	VERB	<LOQ	<LOQ	<LOQ
	OLEUR	0.5 ± 0.1	10.2 ± 2.6	24.1 ± 4.0
OLE50	HOTyr-hexose *isomer a*	0.05 ± 0.01	1.1 ± 0.1	2.8 ± 0.2
	HOTyr-hexose *isomer b*	0.08 ± 0.01	1.6 ± 0.2	4.1 ± 0.3
	VERB	0.0112 ± 0.0002	0.224 ± 0.004	0.62 ± 0.01
	OLEUR	3.0 ± 0.8	60.5 ± 15.6	155.4 ± 34.4
OLE20	HOTyr-hexose *isomer a*	0.03 ± 0.01	0.8 ± 0.1	2.6 ± 0.1
	HOTyr-hexose *isomer b*	0.05 ± 0.02	1.2 ± 0.2	3.9 ± 0.1
	VERB	0.0090 ± 0.0002	0.179 ± 0.004	0.47 ± 0.03
	OLEUR	3.7 ± 0.9	95.2 ± 26.8	324.1 ± 115.7

### 3.6 Liposomes Preparation

Liposomes as delivery systems of OLEUR and OLEs were formulated with an unsaturated natural phospholipid (DOPC) and Chol in the presence or absence of the cationic galactosylated amphiphile GLT1 (Chart 1), with the aim of enhancing their solubility in water, stability in biological fluids, and bioavailability at the target sites.^62^ OLEUR was selected for inclusion in liposomes because it represents the most abundant polyphenol in the OLEs among those quantified.

Chol in the lipid mixture enhances the stability of the lipid bilayer through the *bilayer-tightening effect*, inducing a dense packing, increasing the orientation order of lipid chains, and then leading to a more compact structure with reduced permeability to water-soluble molecules and increased retention of entrapped cargo.^63^ Moreover, it was added to improve the lipid bilayer stability, mostly in the presence of GLT1 because of its detergent properties and ability to destabilize the lipid bilayer, leading to the formation of micellar aggregates.^42^

The presence of GLT1 as a cationic amphiphile in a lipid bilayer proved to enhance the electrostatic interactions between cationic liposomes developed and the negatively charged bacterial membrane cells, as highlighted in a previous study reported in the literature.^31^ It could also improve the interaction between liposomes and bacteria thanks to a possible specific interaction between its sugar moiety, exposed on the liposomal surface, and lectins or sugar protein transporters because it is known that bacteria express them on the cellular membrane.^64,65^

### 3.7 Liposomes Characterization

The hydrodynamic diameter (*D*_h_), PDI, and ζ potential were investigated for empty and loaded neutral and galactosylated liposomes.

As reported in Table 3, all liposomes show narrow size distributions with diameters between 79 and 120 nm and good PDIs (0.10–0.20), according to the extrusion protocol adopted.

**Table 3:** Physicochemical Features of Empty and Loaded Neutral and Galactosylated Liposomes (10 mM Total Lipids) in PBS (pH 7.4)

formulation	composition	*D*_h_ (nm)	PDI	ζ potential (mV)	EE (%)
**1**	DOPC/Chol (8.0:2.0)	119 ± 2	0.10 ± 0.02	–3 ± 2	
**1a**	DOPC/Chol/OLEUR (8.0:2.0:1.25)^a^	100 ± 2	0.12 ± 0.01	–10 ± 1	73 ± 2
**1b**	DOPC/Chol/OLE100 (8.0:2.0)^a^	102 ± 1	0.18 ± 0.01	–10 ± 1	26 ± 4
**1c**	DOPC/Chol/OLE50 (8.0:2.0)^a^	111 ± 1	0.18 ± 0.01	–10 ± 5	32 ± 5
**1d**	DOPC/Chol/OLE20 (8.0:2.0)^a^	120 ± 1	0.20 ± 0.01	–10 ± 5	43 ± 7
**2**	DOPC/Chol/GLT1 (7.0:2.0:1.0)	94 ± 2	0.12 ± 0.01	16 ± 1	
**2a**	DOPC/Chol/GLT1/OLEUR (7.0:2.0:1.0:1.25)^b^	79 ± 1	0.14 ± 0.01	14 ± 3	75 ± 5
**2b**	DOPC/Chol/GLT1/OLE100 (7.0:2.0:1.0)^b^	93 ± 1	0.15 ± 0.01	10 ± 2	36 ± 6
**2c**	DOPC/Chol/GLT1/OLE50 (7.0:2.0:1.0)^b^	94 ± 1	0.16 ± 0.01	10 ± 3	51 ± 7
**2d**	DOPC/Chol/GLT1/OLE20 (7.0:2.0:1.0)^b^	119 ± 1	0.20 ± 0.01	9 ± 1	36 ± 6

a The [phenol]/[total lipids] molar ratio at the beginning of the preparation is 1:8.

b The OLE/total lipids ratio at the beginning of the preparation is 1:1 (w/w).

It is worth highlighting the slight reduction in size for galactosylated formulations compared to the neutral ones, both for OLEUR and OLEs encapsulation, due to the arrangement caused by GLT1 within the DOPC/Chol bilayer. The only exception is represented by liposomes of formulation **2d**, which show dimensions similar to those of the liposomes of the corresponding formulation lacking GLT1 (**1d**). In particular, liposomes of these two formulations (**1d** and **2d**) are characterized by the highest values of hydrodynamic diameters among all formulations studied. This is probably due to the different polyphenol composition of OLE20 with respect to the other OLEs, which could arrange in a different way into the lipid bilayer after their entrapment.

Furthermore, OLEUR encapsulation in neutral and galactosylated liposomes induced a decrease in the average size of liposomes compared to the reference empty formulations. In fact, either of these liposomes (**1a** and **2a**) display the lowest values of hydrodynamic diameters between all formulations studied. This behavior may suggest that OLEUR changes the normal conformation of both lipid bilayers developed.^66^

With the aim to investigate the surface charge of liposomes, the ζ-potential values were determined by electrophoretic mobility measurements using PALS. According to the reported results, DOPC/Chol empty liposomes feature a small negative ζ-potential value due to exposure of the phosphocholine phosphate groups, although the net charge of the zwitterionic phospholipid polar head is zero. The inclusion of OLEUR or OLEs in DOPC/Chol liposomes induced a slight decrease in the ζ-potential values, which become more negative, probably due to localization of the biocompounds loaded onto the membrane surfaces, which could interact with the polar headgroups of DOPC through the formation of hydrogen bonds.^67^

Instead, DOPC/Chol/GLT1-based liposomes, both empty and loaded, exhibit a positive and quite high ζ potential, with a slight decrease in value when OLEUR or OLEs are entrapped, thus reducing the probability of aggregation phenomena accountable for the physical instability of liposomes.^68^ Moreover, the positive ζ-potential values recorded represent indirect evidence of GLT1 inclusion within the lipid bilayer.

The EE (%) of OLEUR in liposomes was evaluated by UPLC measurements. OLEUR-loaded liposomes feature quite high EE %, with OLEUR molar concentrations of 0.92 and 1.0 mM for neutral and galactosylated liposomes, respectively.

Regarding the OLEs-loaded liposomes, EE % was assessed by Folin–Ciocalteu assay. Based on the results reported in Table 3, there is a slight increase of EE % values for all OLEs loaded in galactosylated liposomes (**2b**–**2d**) compared to the neutral ones (**1b**–**1d**). Although EE % appears to be low for OLEs-loaded liposomes, the TPC is actually quite high. This is because the total amount of encapsulated phenols remains significant due to the large quantities of extract used during the loading process into the liposomes, with a lipid-to-extract weight ratio of 1:1.

Furthermore, the EE % values and relative amounts of main polyphenols of OLE100, OLE50, and OLE20 encapsulated in neutral (**1b**–**1d**) and galactosylated (**2b**–**2d**) liposomes were evaluated by UPLC measurements, and the results are reported in Tables 4 and 5. Compared to the quantitative analysis performed on unencapsulated OLEs (Table 2), the relative ratio between polyphenols in free OLEs and loaded in liposomes is only slightly modified, except for VERB, for which its relative encapsulated amount was not detectable (<LOD). It should be noted that olive leaves, and, consequently, the OLEs produced, are already poor in VERB content.

**Table 4:** Entrapment Efficiencies (EE %) of HOTyr-hexose *Isomer a* and *Isomer b*, VERB, and OLEUR Entrapped in OLEs-Loaded Liposomes^a^

	EE (%)					
	**1b**	**2b**	**1c**	**2c**	**1d**	**2d**
HOTyr-hexose *isomer a*	61	63	66	73	65	76
HOTyr-hexose *isomer b*	53	57	57	65	49	66
VERB	nd	nd	nd	nd	nd	nd
OLEUR	68	72	61	72	48	70

a **1** = DOPC/Chol liposomes; **2** = DOPC/Chol/GLT1 liposomes; **b** = OLE100; **c** = OLE50; **d** = OLE20; nd = not determined.

**Table 5:** Relative Amounts (μg/mL) of HOTyr-hexose *Isomer a* and *Isomer b*, VERB, and OLEUR Entrapped in OLEs-Loaded Liposomes^a^

	relative amounts (μg/mL)					
	**1b**	**2b**	**1c**	**2c**	**1d**	**2d**
HOTyr-hexose *isomer a*	3.69 ± 0.01	3.724 ± 0.004	4.05 ± 0.02	4.09 ± 0.01	3.24 ± 0.01	3.59 ± 0.02
HOTyr-hexose *isomer b*	7.56 ± 0.03	7.72 ± 0.02	9.72 ± 0.06	10.13 ± 0.03	6.36 ± 0.03	7.89 ± 0.08
VERB	nd	nd	nd	nd	nd	nd
OLEUR	56.5 ± 0.4	39.3 ± 0.4	294.2 ± 2.2	336.2 ± 0.8	406.6 ± 1.5	621.8 ± 4.9

a **1** = DOPC/Chol liposomes; **2** = DOPC/Chol/GLT1 liposomes; **b** = OLE100; **c** = OLE50; **d** = OLE20; nd = not determined.

#### 3.7.1 Storage Stability

The storage stability over time at 4 °C of all liposomes developed was investigated by dynamic light scattering (DLS) measurements, checking the size and PDI for 90 days (Table S3).

A great physical stability was observed for both OLEUR-loaded liposomes (**1a** and **2a**), highlighting no changes in the dimensions and PDI during all of the storage time investigated.

OLEs-loaded DOPC/Chol liposomes (**1b**–**1d**) turn out to be less stable than the corresponding DOPC/Chol/GLT1 liposomes (**2b**–**2d**), with a slight increase in the dimensions and PDIs during storage. Except for liposomes **2d**, which experienced an increment in the size and PDI values up to 90 days, all of the other cationic galactosylated liposomes did not highlight any evidence of instability during their storage; this is in accordance with their quite high ζ potential, which reduces the probability of aggregation phenomena.

#### 3.7.2 pH Stability

Because liposomes are thermodynamically unstable systems, lipid vesicles could undergo degradation or aggregation under environmental shock conditions such as pH variation. In particular, with regard to polyphenol-loaded liposomes, the pH is a noticeable factor affecting the polyphenol positions inside the lipid bilayer. In an acidic environment, phenolic hydroxyl groups are protonated, and, consequently, polyphenols tend to locate in the hydrophobic region of liposomes, while in an alkaline environment, polyphenols are deprotonated and they prefer to interact with polar headgroups at the lipid bilayer–water interface.^69^

In the case of a potential oral administration *in vivo*, liposomes can experience significant pH variation in the environment around them. Therefore, the stability of OLEUR- and OLEs-loaded liposomes to pH variations was evaluated by DLS measurements, checking vesicle size and PDI at different pH values. To this purpose, the pH of a liposomes solution was adjusted by adding an aqueous solution of HCl or NaOH to mimic those of the human digestive system, in particular pH 5–7 for mouth, pH 1–5 for stomach, pH 6–7.5 for small intestine, and pH 5–8.5 for colon. For these specific values of the pH, the vesicle size and PDI of liposomes were checked after a time corresponding to that of the physiological transit in the tract of the digestive system that we are mimicking. All data collected (Table S4) at different pH values were compared to those obtained at pH = 7.4 (reference value, data reported in blue), corresponding to the physiological pH of blood; for all liposomes produced, great stability to pH variation was observed without significant changes in the dimensions and PDI values.

#### 3.7.3 *In Vitro* Release Study

With the aim of evaluating the releasing profiles of OLEUR and OLEs from liposomes, an *in vitro* study was assessed by a dialysis method.

**Figure 1:**
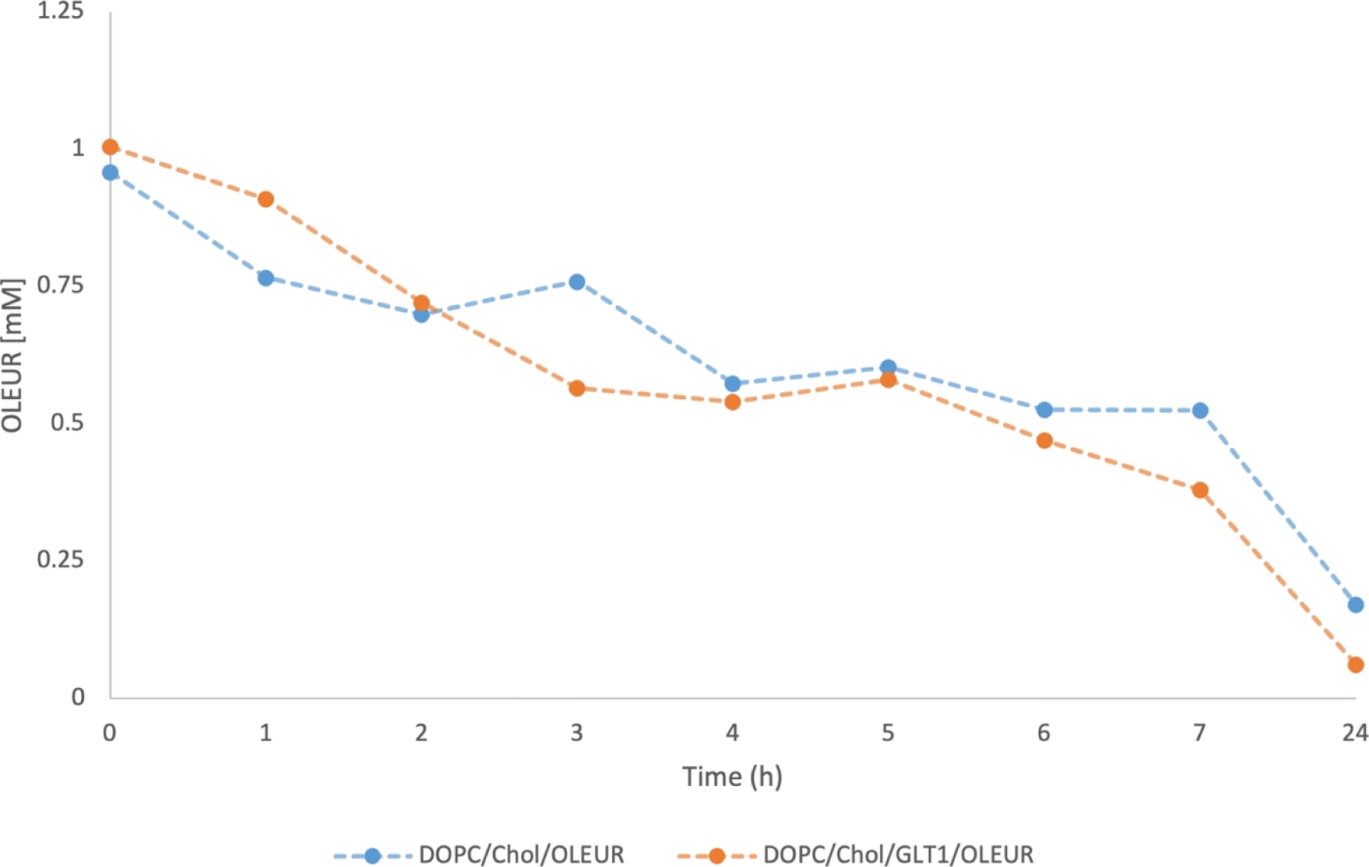
OLEUR content still loaded in DOPC/Chol liposomes (blue dots) and DOPC/Chol/GLT1 liposomes (orange dots) at a specific time over a period of 24 h under forced-release conditions.

The release of OLEUR from liposomes of formulations **1a** and **2a** was examined by UPLC analysis, determining the OLEUR content still encapsulated in neutral and galactosylated liposomes at a specific time over a period of 24 h under forced-release conditions (Figure 1).

Both release curves highlighted a similar trend characterized by a progressive OLEUR reduction content during the first 7 h, and after 24 h, final OLEUR leakages of ∼90% and ∼80% from DOPC/Chol/GLT1 and DOPC/Chol liposomes were observed, respectively. The higher content of OLEUR released from DOPC/Chol/GLT1 liposomes may be related to the detergent properties and destabilizing capacity of GLT1, which could induce a higher release of OLEUR from the DOPC/Chol/GLT1 lipid bilayer compared to the DOPC/Chol lipid bilayer.

The *in vitro* release study of phenolic compounds of OLE100, OLE50, and OLE20 from neutral (**1b**–**1d**) and galactosylated (**2b**–**2d**) liposomes was evaluated over time by Folin–Ciocalteu assay, determining the TPC still encapsulated in liposomes over a period of 24 h.

**Figure 2:**
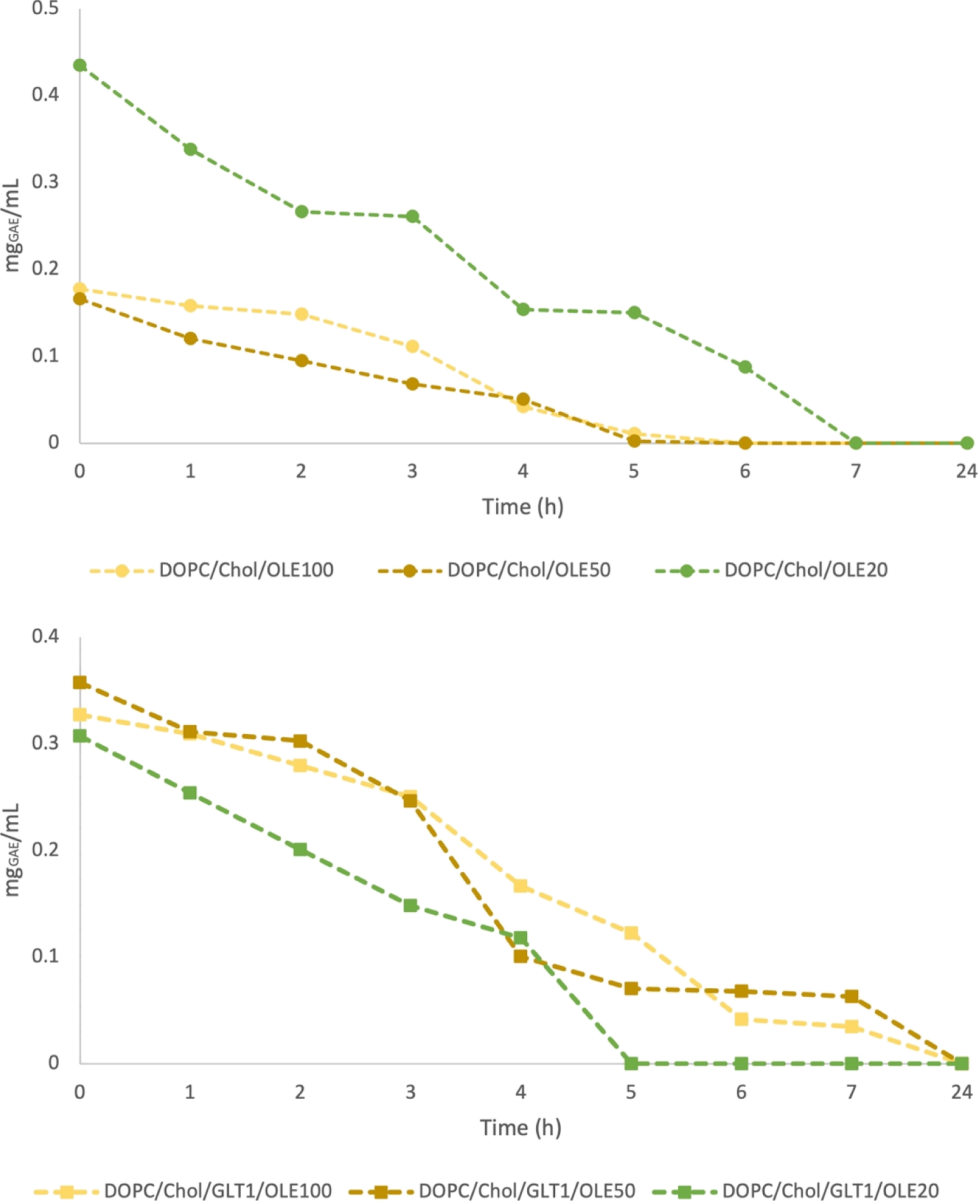
TPC still encapsulated in OLEs-loaded neutral (dots) and galactosylated liposomes (squares) over time under forced-release conditions.

As shown in Figure 2, the release of ∼50% of polyphenols occurred in the first 3–4 h for all extract-loaded liposomes, with a complete cargo release within 5–6 h from DOPC/Chol liposomes and 7–24 h from DOPC/Chol/GLT1 liposomes, respectively.

### 3.8 Antimicrobial Activity

The antimicrobial activity of HOTyr, VERB, OLEUR, and OLEs, in free form and loaded in neutral or galactosylated liposomes, was investigated against two strains of *S. aureus*, ATCC 25923 (wild-type strain) and ATCC 33591 (MRSA), determining the MIC and MBC with the microdilution method.

**Chart 2:**
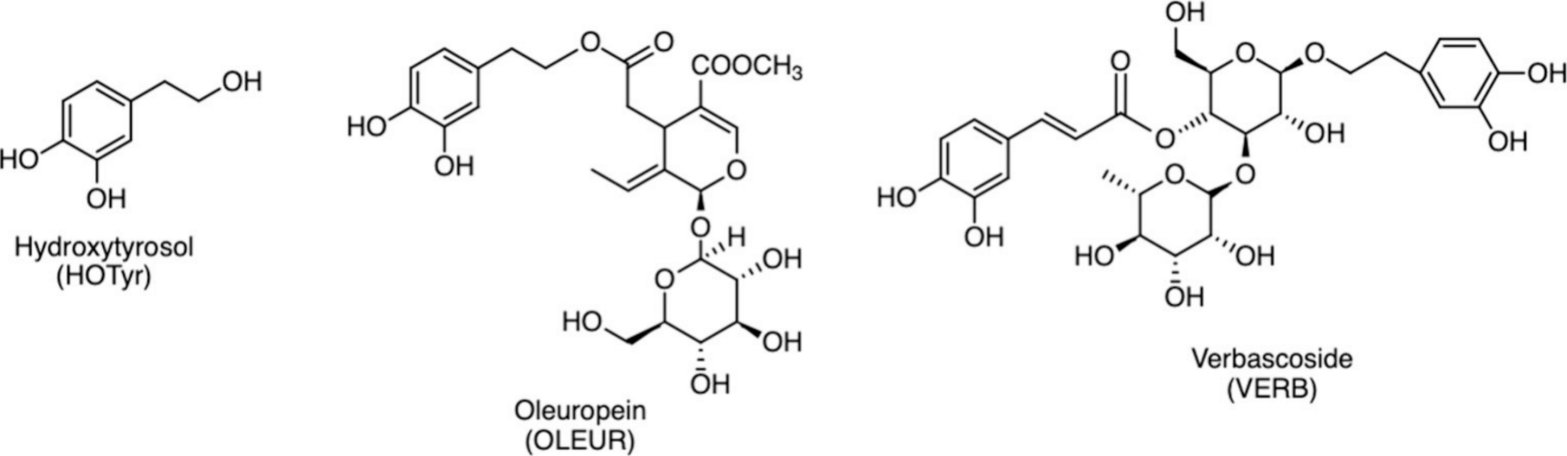
Molecular Structures of Polyphenols under Investigation

The molecular structures of the single polyphenols under investigation are reported in Chart 2.

All of the polyphenols investigated possess interesting biological properties: HOTyr has been one of the most widely studied polyphenols in the last years due to its antiinflammatory, antithrombotic, anticancer, antioxidant, and antimicrobial properties;^70,71^ VERB is a phenylpropanoid glycoside highly widespread in the plant kingdom featuring antimicrobial, antiinflammatory, anticancer, antioxidant, and neuroprotective properties;^72^ OLEUR belongs to the secoiridoids family with antiinflammatory, antioxidant, hepatoprotective, neuroprotective, and antiviral properties and antimicrobial activity affecting both Gram-positive and Gram-negative bacteria.^73,74^

The antimicrobial activity of HOTyr as such was evaluated because of the lack of HOTyr-hexose isomers as reference standards.

The MIC and MBC values of HOTyr, VERB, and OLEUR, tested in free form against both bacterial strains, are reported in Table 6 and expressed as micrograms of compound per milliliter (μg/mL) and as absolute concentration (μM).

**Table 6:** Antimicrobial Activity of HOTyr, VERB, and OLEUR on ATCC 25923 and ATCC 33591

	*S. aureus* (ATCC 25923)				MRSA (ATCC 33591)			
	MIC		MBC		MIC		MBC	
compound	μg/mL	μM	μg/mL	μM	μg/mL	μM	μg/mL	μM
HOTyr	18	117	20	130	19	123	21	136
VERB	48	77	51	82	37	59	51	82
OLEUR	75	138	90	167	84	155	94	174

VERB turned out to be the most active polyphenol among those tested with MICs of 77 μM against *S. aureus* wild type and 59 μM against MRSA, thus highlighting a higher inhibitory activity against the antibiotic-resistant strain compared to the wild-type one. Instead, the MBC value of VERB for both bacterial strains was 82 μM. Although OLEUR is the most abundant polyphenol identified in our extracts, it is the least active among those investigated, with MIC values of 138 μM against the wild-type strain and 155 μM against the resistant strain. HOTyr shows an intermediate antimicrobial activity between VERB and OLEUR, which resulted in higher activity on the wild-type strain compared to MRSA (MIC = 117 μM and MBC = 130 μM for *S. aureus* wild type; MIC = 123 μM and MBC = 136 μM for MRSA).

The MIC and MBC values of OLE100, OLE50, and OLE20, tested in free form, are reported in Table 7 and expressed as milligrams of dry extract per milliliter (mg_extract_/mL).

**Table 7:** Antimicrobial Activity of the OLEs on ATCC 25923 and ATCC 33591

	*S. aureus* (ATCC 25923)		MRSA (ATCC 33591)	
extract	MIC (mg_extract_/mL)	MBC (mg_extract_/mL)	MIC (mg_extract_/mL)	MBC (mg_extract_/mL)
OLE100	0.24	0.29	0.24	0.31
OLE50	0.15	0.18	0.14	0.18
OLE20	0.18	0.19	0.16	0.18

The hydroalcoholic extracts OLE50 and OLE20 show higher activity than the aqueous extract OLE100, with quite similar MIC and MBC values. In particular, MIC values recorded in both cases are lower against ATCC 33591 than ATCC 25923, suggesting a stronger inhibitory effect on the resistant strain than on the wild type. Instead, the MBC values of OLE50 and OLE20 are mainly the same for both bacterial strains investigated.

Although OLE100 is the least active extract investigated, its antimicrobial activity is higher than that expected for its OLEUR content (6.5 times less abundant than in OLE50 and 13.5 times less abundant than in OLE20; Table 2). Nevertheless, its content in HOTyr-hexose *isomer a* and *isomer b* turned out to be essentially the same as those of OLE20 and OLE50; therefore, this probably contributes to partially decreasing the loss of antimicrobial activity of OLE100.

Moreover, it is worth noting that the concentrations at which the OLEs were active correspond to the amounts of HOTyr-hexose *isomer a* and *isomer b*, VERB, and OLEUR considerably lower than those of MIC and MBC determined for the individual compounds, hence highlighting a possible synergistic effect between the bioactive compounds inside the extracts tested. For example, if we consider OLE50, its MIC value is 0.15 mg_extract_/mL against ATCC 25923, and the concentration of OLEUR present in this amount of OLE50 is 23.3 μg/mL, which is 3.2 times lower than the MIC found for free OLEUR (75 μg/mL). A similar consideration can be made for all of the other compounds identified and investigated.

Afterward, the effect of encapsulation in DOPC/Chol and DOPC/Chol/GLT1 liposomes on OLEUR antimicrobial activity was evaluated. The MIC and MBC values of OLEUR loaded in both formulations are reported in Table 8, and the results are expressed both as micrograms of compound per milliliter (μg/mL) and as absolute concentration (μM).

**Table 8:** Antimicrobial Activity of OLEUR Free and Loaded in Liposomes on ATCC 25923 and ATCC 33591

		*S. aureus* wild type (ATCC 25923)				MRSA (ATCC 33591)			
		MIC		MBC		MIC		MBC	
compound	formulation^a^	μg/mL	μM	μg/mL	μM	μg/mL	μM	μg/mL	μM
OLEUR	–	75	139	90	167	84	155	94	174
	**1a**	111	205	131	242	115	213	131	242
	**2a**	107	198	129	239	129	233	146	270

a **1** = DOPC/Chol liposomes; **2** = DOPC/Chol/GLT1; – = OLEUR in free form.

The inclusion of OLEUR in DOPC/Chol and DOPC/Chol/GLT1 liposomes did not highlight any improvement in terms of the antimicrobial activity on both bacteria investigated; in fact, higher MIC and MBC values were obtained compared to those collected for OLEUR tested in free form. Nevertheless, considering all of the beneficial effects derived from the inclusion of OLEUR in liposomes on its pharmacokinetic features (stability, release profile, bioavailability, etc.), the higher values of MIC and MBC determined for OLEUR after encapsulation should not be considered as a negative result. Moreover, OLEUR loaded in both types of liposomes, **1a** and **2a**, proved to be more active against the wild-type strain compared to MRSA.

Finally, the effect of inclusion in neutral and galactosylated liposomes on the antimicrobial activity of OLEs has also been investigated, determining their MIC and MBC values against the two selected *S. aureus* strains. Because we ascribe the antimicrobial activity of OLEs to the polyphenols present in the extracts and we cannot quantify their total amount when encapsulated, we assumed that it was reasonable to report MIC and MBC values of both free and encapsulated extracts as micrograms of gallic acid equivalents per milliliter (μg_GAE_/mL, assessed by Folin–Ciocalteau assay), and this approach allows for meaningful comparisons of the antimicrobial activity.^9^ The results obtained on ATCC 25923 and ATCC 33591 are reported in Tables 9 and 10, respectively.

**Table 9:** Antimicrobial Activity of OLEs Free and Loaded in Liposomes on ATCC 25923^a^

		*S. aureus* wild type (ATCC 25923)			
		MIC		MBC	
extract	formulation	mg_extract_/mL	μg_GAE_/mL	mg_extract_/mL	μg_GAE_/mL
OLE100	–	0.24	12.8	0.29	15.4
	**1b**	nd	30.5	nd	31.2
	**2b**	nd	8	nd	8.2
OLE50	–	0.15	9.3	0.18	11.2
	**1c**	nd	34.4	nd	41.9
	**2c**	nd	10.8	nd	14.1
OLE20	–	0.18	11.7	0.19	12.4
	**1d**	nd	36	nd	37.6
	**2d**	nd	8.6	nd	9.6

a **1** = DOPC/Chol liposomes; **2** = DOPC/Chol/GLT1 liposomes; **b** = OLE100; **c** = OLE50; **d**= OLE20; – = OLE in free form; nd = not determined.

**Table 10:** Antimicrobial Activity of OLEs Free and Loaded in Liposomes on ATCC 33591^a^

		MRSA (ATCC 33591)			
		MIC		MBC	
extract	formulation	mg_extract_/mL	μg_GAE_/mL	mg_extract_/mL	μg_GAE_/mL
OLE100	–	0.24	12.8	0.31	16.6
	**1b**	nd	34.8	nd	35.5
	**2b**	nd	8.5	nd	8.9
OLE50	–	0.14	8.7	0.18	11.2
	**1c**	nd	75.5	nd	78.2
	**2c**	nd	10.8	nd	11.2
OLE20	–	0.16	10.4	0.18	11.7
	**1d**	nd	57.3	nd	58.9
	**2d**	nd	8.9	nd	9.1

a **1** = DOPC/Chol liposomes; **2** = DOPC/Chol/GLT1 liposomes; **b** = OLE100; **c** = OLE50; **d** = OLE20; - = OLE in free form; n.d. = not determined.

OLEs in neutral liposomes (**1b**–**1d**) did not lead to any improvement in terms of antimicrobial activity against both bacterial strains, hence resulting in higher MIC and MBC values than those collected for OLEs in free form. With regard to galactosylated liposomes, we obtained comparable or slightly increased antimicrobial activity for all loaded OLEs with respect to the free ones, without any substantial differences in the MIC and MBC values among the bacterial strains investigated. In particular, the encapsulation in galactosylated liposomes displayed a positive effect on the antimicrobial activity of OLE100 and OLE20, while any improvement was observed for OLE50 activity, although it is not as detrimental as in the case of OLEs encapsulation in neutral liposomes. OLEs antimicrobial activity improvement assessed after encapsulation in DOPC/Chol/GLT1 liposomes is probably related to the presence of GLT1 inside the lipid bilayer, which proved to enhance the interaction between liposomes and bacteria through the electrostatic interaction of cationic liposomes with the negatively charged bacteria. On the other hand, the interaction between galactose residues, exposed on the liposome surface, and lectins or sugar transporters, expressed by the bacterial membrane, could lead to the better diffusion and interaction of the active compounds released from the lipid bilayer across the bacterial cell walls, which, coupled with the synergistic effect of OLE polyphenols released, leads to an increase in the antimicrobial activity.

The activity of DOPC/Chol and DOPC/Chol/GLT1 empty liposomes was also evaluated against both bacterial strains, and there was no evidence of antimicrobial activity caused by the lipidic components of liposomes in both cases.

## 4 Conclusions

The investigation here reported represents an example of a circular economy approach toward the valorization of agrifood waste. *O. europaea* leaves, a byproduct of the olive oil chain that poses both economic and environmental challenges for producers, were used to produce extracts with antibacterial activity investigated against two strains of *S. aureus*, ATCC 25923 (wild-type strain) and ATCC 33591 (MRSA).

All of the extracts exhibited significant antimicrobial activity against both strains under investigation, potentially due to a synergistic effect among the bioactive compounds in the tested phytocomplexes. The observed synergistic effect of olive leaf polyphenols not only enhances their efficacy in treating bacterial infections but also may significantly help in preventing the development of antibiotic resistance and extend the bioactive compounds lifetime.

Furthermore, encapsulating olive leaf polyphenols in DOPC/Chol/GLT1 liposomes, besides improving their solubility, stability, and bioavailability, does not affect their antimicrobial activity, with the exhibition of comparable or slightly enhanced activity against both bacterial strains compared to the free extracts.

These findings pave the way for new strategies in treating drug-resistant infections, a major concern in the era of antibiotic resistance, by exploiting the synergistic effects of polyphenols obtained from botanical extracts delivered through functionalized liposomes as targeted drug-delivery systems.

## Supporting Information Available

Analytical parameters of the method proposed (Table S1), preliminary data of OLE50 extraction carried out at different times (Table S2), storage stability of liposomes under investigation (Table S3), pH stability of liposomes under investigation (Table S4), chromatographic profile of the standards mixture used for the identification of phenolic compounds in the extracts (Figure S1), chromatographic profiles of OLE100, OLE50, and OLE20 (Figure S2), ESI-MS detection in negative scanning mode for *m*/*z* 315 with the analysis performed on OLE20 (Figure S3), UV–vis spectra of hydroxytyrosol-hexose *isomer a* (Figure S4), hydroxytyrosol-hexose *isomer b* (Figure S5), verbascoside (Figure S6), and oleuropein (Figure S7), and MS spectra of hydroxytyrosol-hexose *isomer a* (Figure S8), hydroxytyrosol-hexose *isomer b* (Figure S9), verbascoside (Figure S10), and oleuropein (Figure S11) (PDF)

## Abbreviations

Chol: cholesterol
DOPC: 1,2-dioleoyl-*sn*-glycero-3-phosphocholine
EE: entrapment efficiency
GLT1: cationic galactosylated amphiphile
HOTyr: hydroxytyrosol
MBC: minimum bacteridicial concentration
MIC: minimum inhibitory concentration
MRSA: methicillin-resistant *Staphylococcus aureus*
OLEs: olive leaf extracts
OLEUR: oleuropein
PDI: polydispersity index
VERB: verbascoside
