# Supplementary material for: Encapsulation of Olea europaea Leaf Polyphenols in Liposomes: A Study on Their Antimicrobial Activity to Turn a Byproduct into a Tool to Treat Bacterial Infection
Source: ACS Appl Mater Interfaces. 2024 Dec 4;16(50):68850–63. doi: 10.1021/acsami.4c13302 (PMC11660030; doi:10.1021/acsami.4c13302)
Supplement: Supplementary file 1 — am4c13302_si_001.pdf [file am4c13302_si_001.pdf]

Supporting Information

Encapsulation of *Olea europaea* leaf polyphenols in liposomes: a study on their antimicrobial activity to turn a by-product in a tool to treat bacterial infection

Giuliana Prevete,*[a] Enrica Donati,[a] Anna Paola Ruggiero,[a] Silvia Fardellotti,[a] Laura Lilla,[a] Valentina Ramundi,[a] Isabella Nicoletti,[a] Francesca Mariani,[a] and Marco Mazzonna*[a]

[a] CNR-Institute for Biological Systems (ISB), Territorial Research Area Rome 1, Strada Provinciale 35d, n. 9, 00010 - Montelibretti (Rome), Italy

Corresponding Authors:

*Marco Mazzonna; CNR-Institute for Biological Systems (ISB), Territorial Research Area Rome 1, Strada Provinciale 35d, n. 9, 00010 – Montelibretti (Rome), Italy; email: marco.mazzonna@cnr.it
*Giuliana Prevete, CNR-Institute for Biological Systems (ISB), Territorial Research Area Rome 1, Strada Provinciale 35d, n. 9, 00010 – Montelibretti (Rome), Italy; email: giuliana.prevete@isb.cnr.it

Table S1. Analytical parameters of the method proposed.

**Compounds**	**LOD** (mg/mL)	**LOQ** (mg/mL)	**Calibration curves** (mg/mL)	R^2
Hydroxytyrosol	0.00011	0.00033	y=5000000x-11180	0.9998
Verbascoside	0.00010	0.00036	y=7000000x-6966	0.9997
Oleuropein	0.00038	0.00114	y=909597x-11153	0.9993

Table S2. Preliminary data of OLE50 extraction carried out at different time.

**Time of extraction (min)**	**Yield of extraction (%)**	**Total Phenolic Content (mg_{GAE}/g_{leaves})**	**TEAC t_{1min} ($mmol_{TE}/g_{leaves}$)**	**TEAC t_{4min} ($mmol_{TE}/g_{leaves}$)**
15	31 ± 1	18 ± 2	0.14 ± 0.01	0.15 ± 0.07
30	33 ± 1	22 ± 2	0.19 ± 0.05	0.20 ± 0.05
45	40 ± 2	24 ± 3	0.28 ± 0.02	0.31 ± 0.03

Table S3. Storage stability of liposomes under investigation.

**DOPC/Chol based liposomes**				**DOPC/Chol/GLT1 based liposomes**			
**Formulation**	**Time (day)**	**D_h (nm)**	**PDI**	**Formulation**	**Time (day)**	**D_h (nm)**	**PDI**
**1a**	1	100 ± 2	0.12 ± 0.01	**2a**	1	79 ± 1	0.14 ± 0.01
	30	102 ± 1	0.13 ± 0.01		30	81 ± 1	0.13 ± 0.01
	60	102 ± 1	0.13 ± 0.02		60	81 ± 1	0.15 ± 0.01
	90	102 ± 1	0.14 ± 0.01		90	81 ± 2	0.14 ± 0.01
**1b**	1	100 ± 1	0.23 ± 0.01	**2b**	1	91 ± 1	0.15 ± 0.01
	30	99 ± 1	0.29 ± 0.02		30	93 ± 1	0.14 ± 0.01
	60	104 ± 1	0.29 ± 0.02		60	95 ± 1	0.15 ± 0.01
	90	99 ± 3	0.23 ± 0.07		90	92 ± 1	0.12 ± 0.02
**1c**	1	100 ± 2	0.10 ± 0.01	**2c**	1	90 ± 1	0.12 ± 0.01
	30	107 ± 2	0.17 ± 0.02		30	89 ± 2	0.12 ± 0.02
	60	110 ± 2	0.20 ± 0.02		60	92 ± 1	0.18 ± 0.02
	90	125 ± 1	0.27 ± 0.01		90	93 ± 1	0.16 ± 0.02
**1d**	1	111 ± 2	0.13 ± 0.01	**2d**	1	110 ± 1	0.21 ± 0.01
	30	112 ± 1	0.17 ± 0.01		30	114 ± 3	0.19 ± 0.01
	60	118 ± 3	0.20 ± 0.01		60	117 ± 3	0.18 ± 0.01
	90	136 ± 1	0.26 ± 0.01		90	133 ± 4	0.25 ± 0.01

1 = DOPC/Chol based liposomes; **2** = DOPC/Chol/GLT1 based liposomes; **a** = OLEUR; **b** = OLE100; **c** = OLE50; **d** = OLE20.

Table S4. pH stability of liposomes under investigation.

DOPC/Chol liposomes				DOPC/Chol/GLT1 liposomes			
**Formulation**	**pH**	**D_h (nm)**	**PDI**	**Formulation**	**pH**	**D_h (nm)**	**PDI**
	2.9	96 ± 1	0.11 ± 0.01		2.9	84 ± 1	0.12 ± 0.01
	5.7	96 ± 1	0.09 ± 0.01		5.7	84 ± 1	0.12 ± 0.01
**1a**	6.4	96 ± 1	0.11 ± 0.01	**2a**	6.4	85 ± 1	0.13 ± 0.01
	7.4	94 ± 1	0.11 ± 0.02		7.4	87 ± 1	0.15 ± 0.01
	8.1	94 ± 1	0.10 ± 0.03		8.1	84 ± 1	0.12 ± 0.01
	2.9	104± 1	0.31 ± 0.01		2.9	89 ± 1	0.16 ± 0.01
	5.7	101 ± 2	0.12 ± 0.01		5.7	91 ± 1	0.15 ± 0.01
**1b**	6.4	98 ± 1	0.13 ± 0.02	**2b**	6.4	90 ± 1	0.18 ± 0.01
	7.4	100 ± 1	0.17 ± 0.01		7.4	92 ± 1	0.19 ± 0.01
	8.1	95 ± 1	0.23 ± 0.01		8.1	88 ± 2	0.17 ± 0.01
	2.9	112 ± 1	0.22 ± 0.01		2.9	92 ± 1	0.15 ± 0.01
	5.7	98 ± 1	0.16 ± 0.01		5.7	92 ± 1	0.16 ± 0.01
**1c**	6.4	97 ± 1	0.15 ± 0.01	**2c**	6.4	92 ± 1	0.15 ± 0.01
	7.4	103 ± 1	0.17 ± 0.01		7.4	94 ± 1	0.16 ± 0.01
	8.1	109 ± 2	0.18 ± 0.01		8.1	93 ± 1	0.17 ± 0.01
	2.9	123 ± 2	0.22 ± 0.02		2.9	115 ± 2	0.16 ± 0.01
	5.7	118 ± 1	0.21 ± 0.01		5.7	117 ± 1	0.16 ± 0.01
**1d**	6.4	121 ± 2	0.22 ± 0.01	**2d**	6.4	115 ± 1	0.17 ± 0.01
	7.4	120 ± 1	0.20 ± 0.01		7.4	119 ± 1	0.20 ± 0.01
	8.1	117 ± 3	0.21 ± 0.01		8.1	116 ± 2	0.17 ± 0.01

1 = DOPC/Chol liposomes; **2** = DOPC/Chol/GLT1 liposomes; **a** = OLEUR; **b** = OLE100; **c** = OLE50; **d** = OLE20

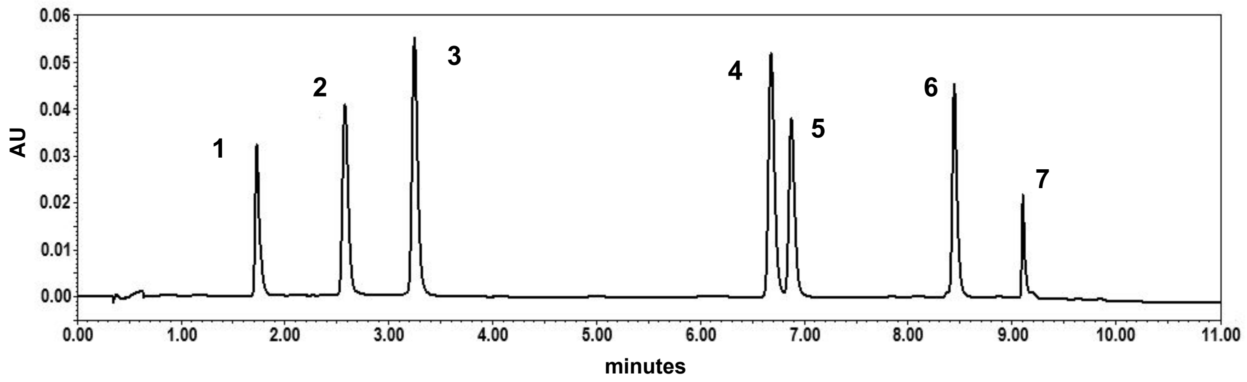

Figure S1. Chromatographic profile of the standards mixture used for the identification of phenolic compounds in the extracts ($\lambda = 280$ nm): 1) Hydroxytyrosol, 2) Tyrosol, 3) Vanillic acid, 4) Luteolin-7-glucoside, 5) Verbascoside, 6) Apigenin-7-glucoside, 7) Oleuropein.

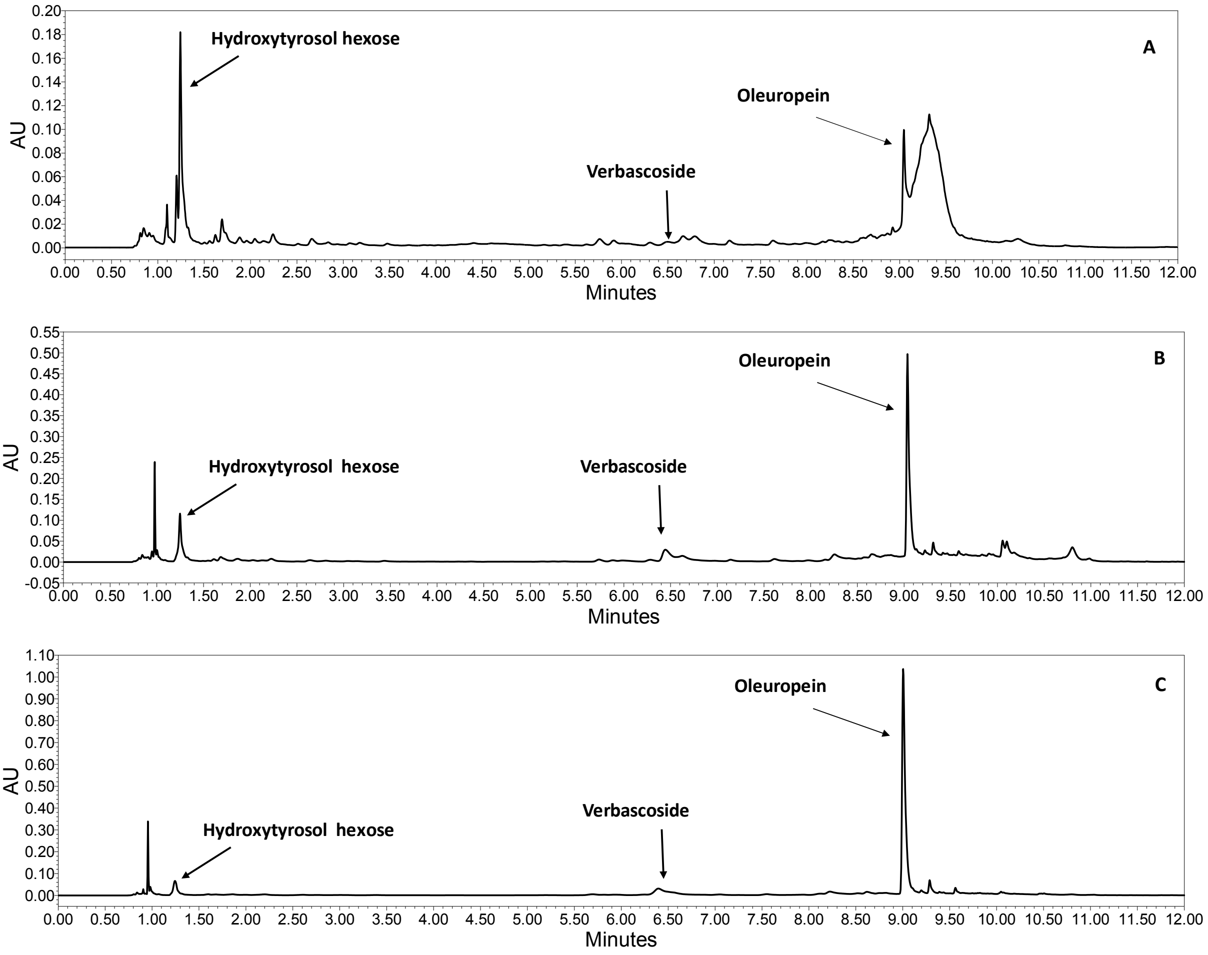

Figure S2. Chromatographic profiles of (A) OLE100, (B) OLE50 and (C) OLE20, detection at 280 nm.

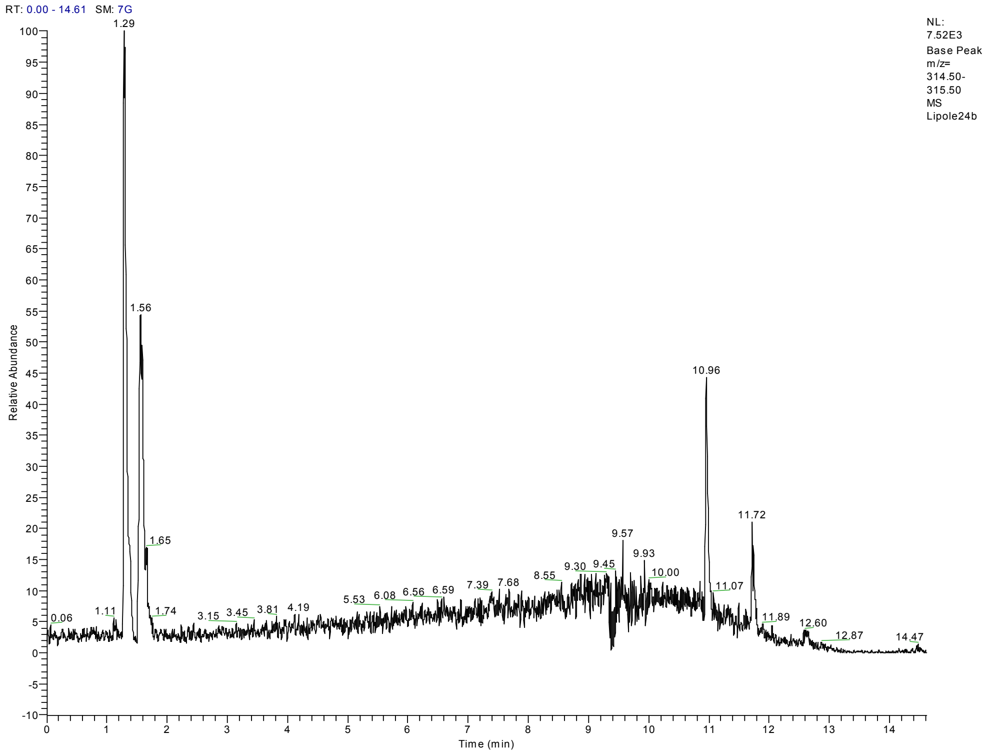

Figure S3. ESI-MS detection in negative scanning mode for *m/z* = 315, analysis performed on OLE20.

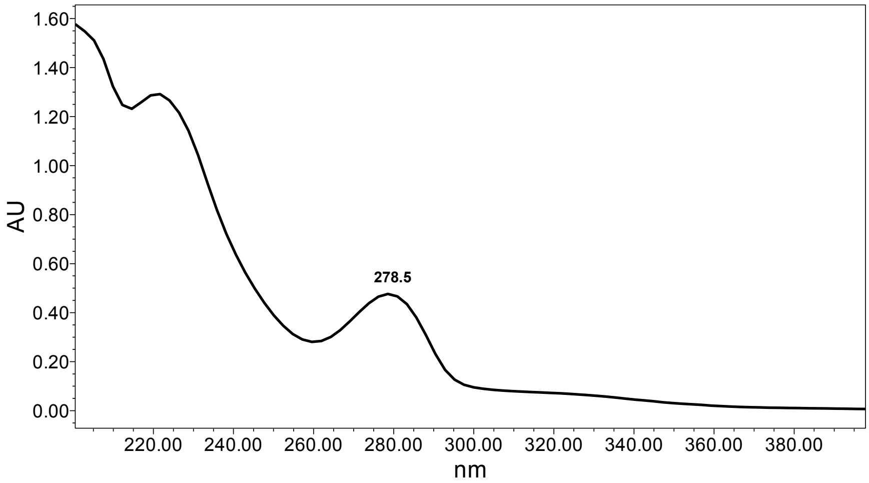

Figure S4. UV-Vis spectra of Hydroxytyrosol-hexose *isomer a*.

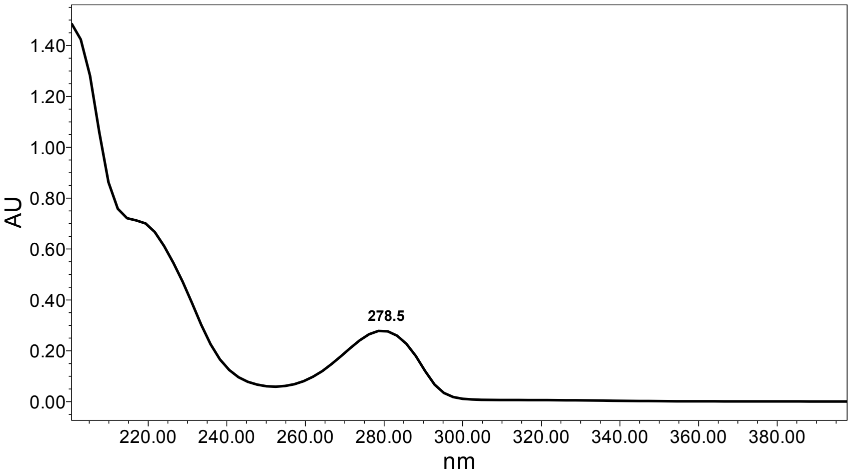

Figure S5. UV-Vis spectra of Hydroxytyrosol-hexose *isomer b*.

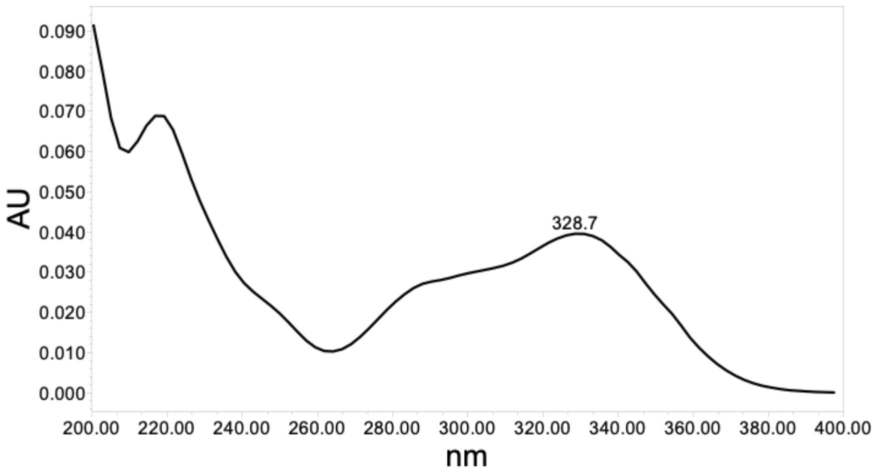

Figure S6. UV-Vis spectra of Verbascoside.

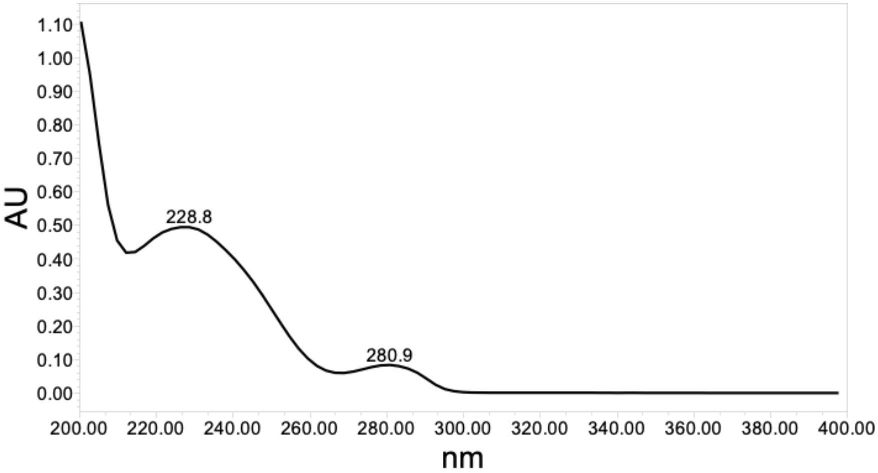

Figure S7. UV-Vis spectra of Oleuropein.

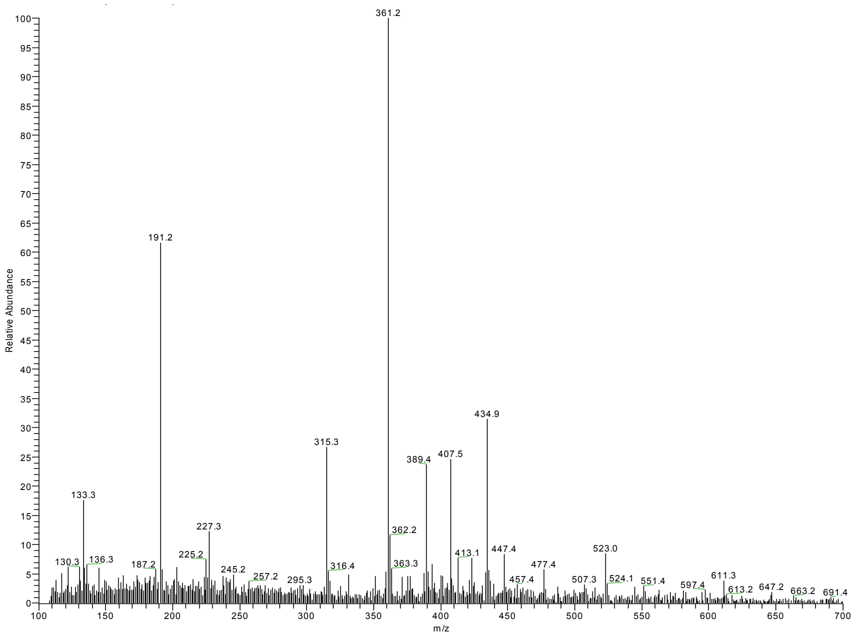

Figure S8. Mass spectrum of Hydroxytyrosol-hexose *isomer a*, molecular ion $[M-H]^-$ *m/z* = 315.

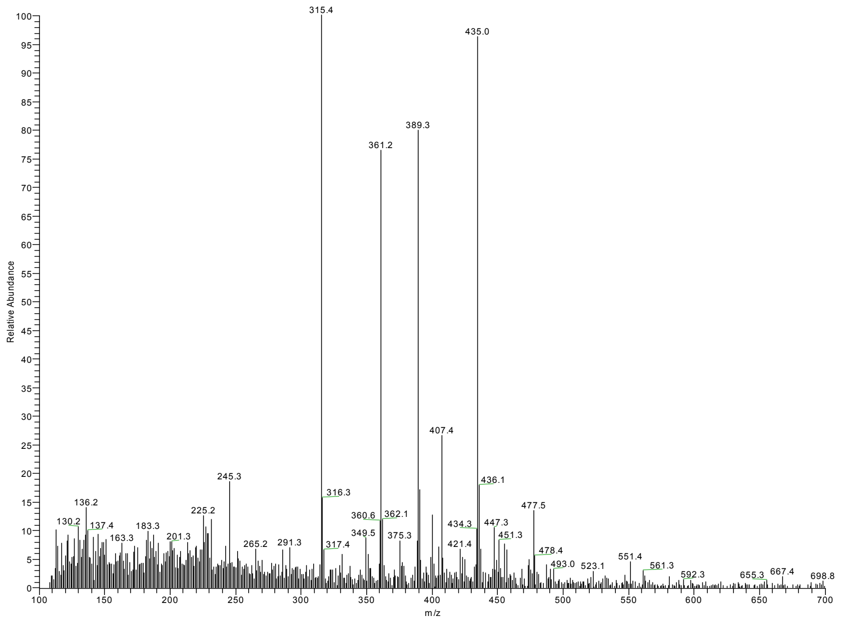

Figure S9. Mass spectrum of Hydroxytyrosol-hexose *isomer b*, molecular ion $[M-H]^-$ m/z = 315.

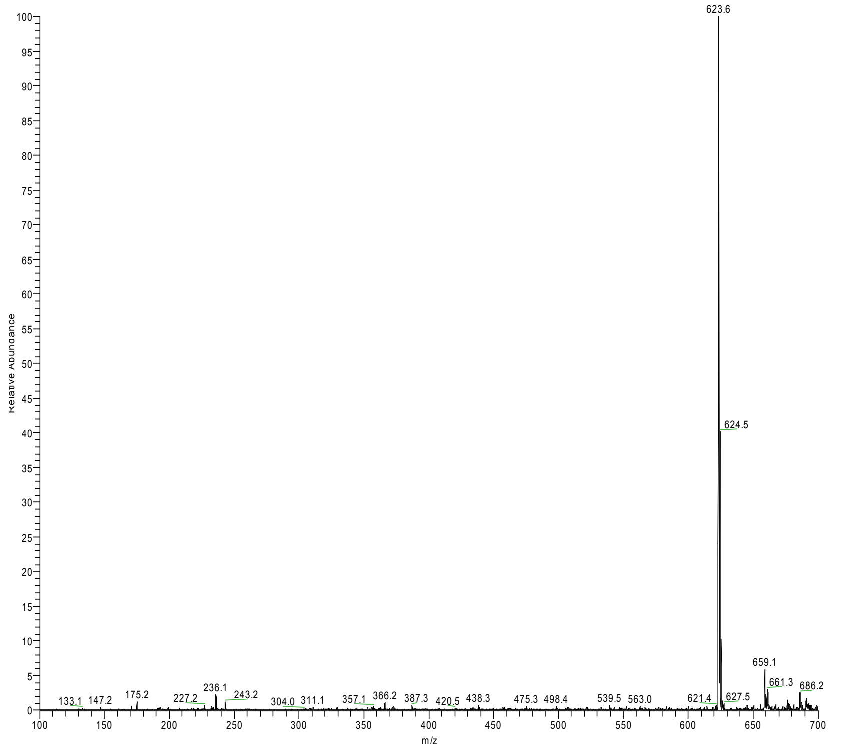

Figure S10. Mass spectrum of Verbascoside, molecular ion $[M-H]^-$ m/z = 623.

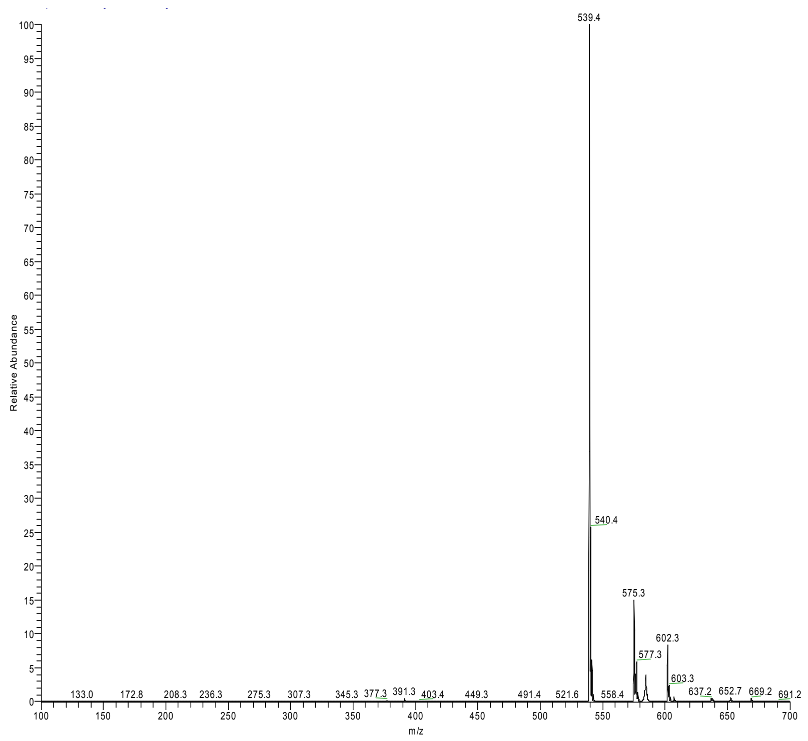

Figure S11. Mass spectrum of Oleuropein, molecular ion $[M-H]^-$ *m/z* = 539.
